# Divergence of bacterial communities in the lower airways of CF patients in early childhood

**DOI:** 10.1371/journal.pone.0257838

**Published:** 2021-10-06

**Authors:** John B. O’Connor, Madison M. Mottlowitz, Brandie D. Wagner, Kathleen L. Boyne, Mark J. Stevens, Charles E. Robertson, Jonathan K. Harris, Theresa A. Laguna

**Affiliations:** 1 Division of Pulmonary and Sleep Medicine, Department of Pediatrics, Ann & Robert H. Lurie Children’s Hospital of Chicago, Chicago, Illinois, United States of America; 2 Department of Pediatrics, School of Medicine, University of Colorado, Anschutz Medical Campus, Aurora, Colorado, United States of America; 3 Department of Biostatistics and Informatics, Colorado School of Public Health, University of Colorado, Anschutz Medical Campus, Aurora, Colorado, United States of America; 4 Department of Pediatrics, Northwestern University Feinberg School of Medicine, Chicago, Illinois, United States of America; 5 Feinberg School of Medicine, Northwestern University, Chicago, Illinois, United States of America; Laurentian University, CANADA

## Abstract

**Rationale:**

Chronic airway infection and inflammation resulting in progressive, obstructive lung disease is the leading cause of morbidity and mortality in cystic fibrosis. Understanding the lower airway microbiota across the ages can provide valuable insight and potential therapeutic targets.

**Objectives:**

To characterize and compare the lower airway microbiota in cystic fibrosis and disease control subjects across the pediatric age spectrum.

**Methods:**

Bronchoalveolar lavage fluid samples from 191 subjects (63 with cystic fibrosis) aged 0 to 21 years were collected along with relevant clinical data. We measured total bacterial load using quantitative polymerase chain reaction and performed 16S rRNA gene sequencing to characterize bacterial communities with species-level sensitivity for select genera. Clinical comparisons were investigated.

**Measurements and main results:**

Cystic fibrosis samples had higher total bacterial load and lower microbial diversity, with a divergence from disease controls around 2–5 years of age, as well as higher neutrophilic inflammation relative to bacterial burden. Cystic fibrosis samples had increased abundance of traditional cystic fibrosis pathogens and decreased abundance of the *Streptococcus mitis* species group in older subjects. Interestingly, increased diversity in the heterogeneous disease controls was independent of diagnosis and indication. Sequencing was more sensitive than culture, and antibiotic exposure was more common in disease controls, which showed a negative relationship with load and neutrophilic inflammation.

**Conclusions:**

Analysis of lower airway samples from people with cystic fibrosis and disease controls across the ages revealed key differences in airway microbiota and inflammation. The divergence in subjects during early childhood may represent a window of opportunity for intervention and additional study.

## Introduction

Chronic airway infection and inflammation resulting in progressive, obstructive lung disease is the leading cause of morbidity and mortality in people with cystic fibrosis (CF) [1]. Previous work has identified traditional pathogens in airway culture (i.e. *Pseudomonas*, *Staphylococcus*, *Haemophilus*, *Burkholderia*, *Stenotrophomonas*, and *Achromobacter*) thought to be drivers of airway damage and lung function decline in CF [1–9]. Culture-independent, molecular approaches have broadened this view from a few individual pathogens to more complex and dynamic polymicrobial communities [3, 10–20]. The role of the airway microbiota in the development of CF lung disease is not well established, especially during childhood. Although new therapies may improve clinical outcomes in CF, airway infection and inflammation will likely remain problematic [21–23]. Understanding the lower airway bacterial communities in CF subjects across the age spectrum, and how they differ from disease control (DC) subjects, can provide insight into the development of CF lung disease and identify potential therapeutic targets.

In infants and young children with CF, culture-independent next-generation sequencing approaches have been useful in describing the upper airway microbiota [12], and previous studies have shown that younger patients have higher diversity which decreases as lung disease progresses [24–26]. Longitudinal analysis of infant samples has identified changes in microbial composition and diversity in the first two years of life [27]; however, these studies focused on upper airway samples which may not adequately reflect the lower airway microbiota [28, 29]. Analysis of bronchoalveolar lavage fluid (BALF) allows for accurate characterization of the lower airway microbiota with minimal upper airway contamination. Through 16S ribosomal RNA (rRNA) gene sequencing, we previously found that organisms not traditionally associated with CF dominate the lower airway of asymptomatic CF infants [30] while others have demonstrated that CF infants tend to have less lower airway diversity and distinct microbiota compared to DCs [31]. Small, single-center studies in children with CF and DCs have also identified traditional and nontraditional CF taxa in the lower CF airway and indicated differences in microbial diversity between groups [32, 33]. While previous work in BALF has suggested that younger people with CF have nontraditional, anaerobic taxa (i.e. *Streptococcus*, *Prevotella*, and *Veillonella)* in their lower airways while older CF subjects are dominated by traditional CF taxa (i.e. *Pseudomonas*, *Staphylococcus*, *and Stenotrophomonas)*, adequate comparison to DC subjects has been limited by smaller sample sizes [34]. With a larger BALF sample set from CF and DC subjects across the age spectrum, we now seek to use a gene-sequencing approach to expand our knowledge of the lower CF airway. Some of the results of these studies have been previously reported in the form of abstracts [35, 36].

## Methods

### Study design and population

CF and DC subjects from the University of Minnesota and University of Colorado were enrolled at the time of clinically indicated flexible bronchoscopy with lavage. DC subjects were identified as those without any confirmed diagnosis of CF. Because healthy pediatric patients cannot undergo bronchoscopies for research purposes alone, no healthy control samples could be included. Subjects consented to have extra BALF sample from the procedure stored in biobanks using institutional review board (IRB)-approved specimen collection protocols. Relevant clinical data was collected from the electronic medical record and the CF Foundation Patient Registry and included height, weight, culture results, lung function measures, and whether the subjects were receiving antibiotics, either oral, inhaled, or intravenous (IV), at the time of sample collection. Written informed consent, adolescent assent, and parental permission with HIPPA (Health Insurance Portability and Accountability Act of 1996) authorization were obtained from subjects and/or legal guardians according to each individual site’s IRB requirements.

### Sample collection and processing

Flexible bronchoscopy and BALF collection were performed in accordance with each institution’s standard-of-care guidelines. During the bronchoscopy procedure, a flexible bronchoscope was passed through the airway via an endotracheal tube or a laryngeal mask airway, and lavages were performed using sterile saline. The area of the lung from which lavage was collected varied depending on the pulmonologist’s selection. Any excess BALF not needed for clinical testing was collected for research. Volume permitting, a portion of neat, unprocessed BALF sample was set aside, and the remaining sample was centrifuged at 250 x g for ten minutes at 4°C, followed by separation of the supernatant and pellet. Samples were all stored at -80°C. Neat samples and pellets were shipped on dry ice overnight to the Children’s Hospital Colorado for quantitative Polymerase Chain reaction (qPCR) and 16s rRNA gene sequencing. Both pellets, processed from higher volume samples, and neat samples, with insufficient volume for processing, were included in the microbiota analysis, as previously performed [32].

### Laboratory assays

Details regarding the laboratory assays are summarized in the supporting information. Briefly, DNA was extracted from BALF samples using the Qiagen EZ1 Advanced automated extraction platform (Qiagen, Valencia, CA, USA) per manufacturer’s instructions. Total bacterial load (TBL) was assessed using the quantitative PCR assay previously described and validated in CF airway samples [37, 38]. Bacterial profiles were determined by amplification of the V1/V2 variable region (27F/338R) of the 16S rRNA gene as previously described [30, 34, 39, 40]. Operational taxonomic units (OTUs) were produced by clustering sequences with identical taxonomic assignments. Negative control samples, consisting of only assay reagents, were included in the sequencing analysis to assess background (S1 and S2 Figs).

### Statistical analysis

Descriptive statistics, including the median and range values, were calculated for all numerical variables. The relative abundance of each taxon was calculated to account for differences in sequencing depth, and Shannon diversity index was used to represent alpha diversity. The relationships between airway microbial measures (TBL and diversity) and age by group (CF or DC) were modelled using cubic B splines. Additional information on the modelling can be found in the supporting information. Functional boxplots were used to describe the distribution of the TBL and diversity curves between groups and across age categories.

Taxa with at least one sequence were considered detected by 16S sequencing. However, to analyze the prevalence of taxa across samples, only the most abundant taxa making up 95% of sample’s overall abundance were included. The remaining less abundant taxa were grouped together into “other taxa” and were not counted toward prevalence.

Chi-squared tests and Fisher’s exact tests, when appropriate, were used to compare categorical variables across disease groups. Wilcoxon rank-based tests were used to compare numerical variables across groups, and Spearman’s correlation coefficients were used to determine the correlation between numerical variables. P-values under 0.05 were considered statistically significant.

## Results

### Study population

Two hundred six BALF samples were collected from 191 subjects (128 DC). Nine samples were neat and 182 were pellets. CF and non-CF study subject demographics and relevant clinical characteristics are summarized in Table 1. Indications and primary diagnoses are summarized in S1 Table.

**Table 1:** Study population.

	DCs (n = 128)	CF (n = 63)	P-value
**Age years, median (range)**	5.2 (0–21.0)	9.7 (0.9–19.7)	<0.001
**<2 years, number (%)**	38 (30%)	3 (5%)	<0.001
**2–5 years, number (%)**	31 (24%)	9 (14%)	
**6–10 years, number (%)**	32 (25%)	25 (40%)	
**11–17 years, number (%)**	17 (13%)	21 (33%)	
**18 years and older, number (%)**	10 (8%)	5 (8%)	
**Female, number (%)**	60 (47%)	41 (65%)	0.018
**Weight (kg), median (range) (data available)**	18.2 (4.2–78.3) (N = 128)	29.0 (13.5–74.7) (N = 49)	<0.001
**Height (cm), median (range) (data available)**	106.0 (51.0–181.0) (N = 120)	132.4 (97.8–175.4) (N = 49)	<0.001
**Genotype, data available**	N/A	N = 62	N/A
** F508del/F508del, number (%)**	N/A	36 (58%)	N/A
** F508del/other, number (%)**	N/A	22 (35%)	N/A
** Other/other, number (%)**	N/A	4 (6%)	N/A
**FEV1** **% predicted, median (range) (data available)**	83.0 (41.0–109.0) (N = 27)	86.5 (35.0–131.0) (N = 50)	0.709
**BALF Cell Counts, data available**	N = 126	N = 53	N/A
** White blood cells, median (range) (data available)**	213 (0–8122) (N = 126)	1312 (66–51500) (N = 53)	<0.001
** Percent Neutrophils, median (range) (data available)**	8 (1–99) (N = 107)	80 (3–99) (N = 51)	<0.001
** Percent Lymphocytes, median (range) (data available)**	9 (1–66) (N = 155)	3 (0–28) (N = 39)	<0.011
**BALF culture results, data available**	N = 128	N = 31	N/A
** Negative, number (%)**	86 (67%)	3 (10%)	<0.001*
** Pseudomonas aeruginosa, number (%)**	2 (2%)	7 (23%)	0.036*
** MSSA, number (%)**	4 (3%)	19 (61%)	<0.001*
** MRSA, number (%)**	0 (0%)	1 (3%)	0.456*
** Haemophilus influenzae, number (%)**	4 (3%)	2 (6%)	0.281*
** Stenotrophomonas maltophilia, number (%)**	0 (0%)	2 (6%)	0.204*
** Achromobacter xylosoxidans, number (%)**	0 (0%)	1 (3%)	0.456*
** Burkholderia cepacia, number (%)**	0 (0%)	0 (0%)	___
** Nontuberculous mycobacteria, number (% positive with NTM testing) (number with test done)**	1 (2%) (N = 52)	2 (3%) (N = 58)	0.398*
**Antibiotic Use number (% of those with data available) (number with data available)**	35 (90%) (N = 39)	28 (45%) (N = 62)	<0.001*

Data are presented as n, median (range) or n (%), unless otherwise stated. CF: Cystic fibrosis; FEV1: Forced expiratory volume in 1 s; BALF: Bronchoalveolar lavage fluid; MSSA: *Methicillin-susceptible Staphylococcus aureus;* MRSA: *Methicillin-resistant Staphylococcus aureus*.

* P-value calculated using Fisher’s exact test

### Sequencing and total bacterial load (TBL)

**Fig 1:**
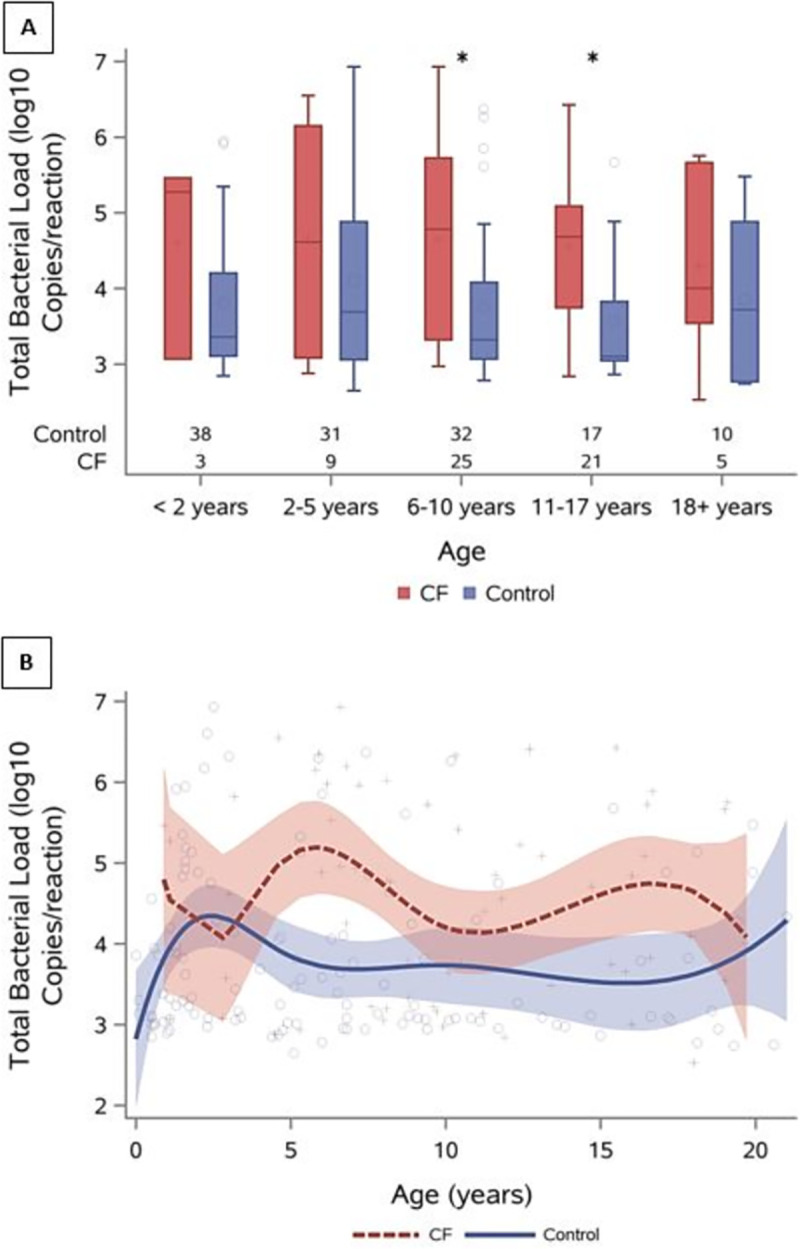
Load across age. (A) Bar plots of TBL in CF and DCs in age groups, sample sizes are displayed along the x-axis and asterisks represent significant differences between CF and DC samples (B) TBL across the age spectrum with cubic B spline fitting (solid line) and 95% confidence intervals (shaded areas). On average BALF samples from CF subjects have higher TBL compared to DC samples, this is more pronounced during early school age and adolescence.

Of the 191 BALF samples, 124 (65%) had sufficient bacterial load for sequencing. Relevant clinical characteristics categorized by sequencing are summarized in S2 Table. CF samples were more likely to have successful amplification, with microbiota sequence data available for 81% of the CF samples and 57% of DCs. TBL was lower in BALF samples that failed sequencing (p<0.001, Wilcoxon rank-based test) (S3 Fig) and generally higher in CF samples, with the difference being more pronounced during early school age (ages 5–8) and adolescence (Fig 1).

### Diversity

**Fig 2:**
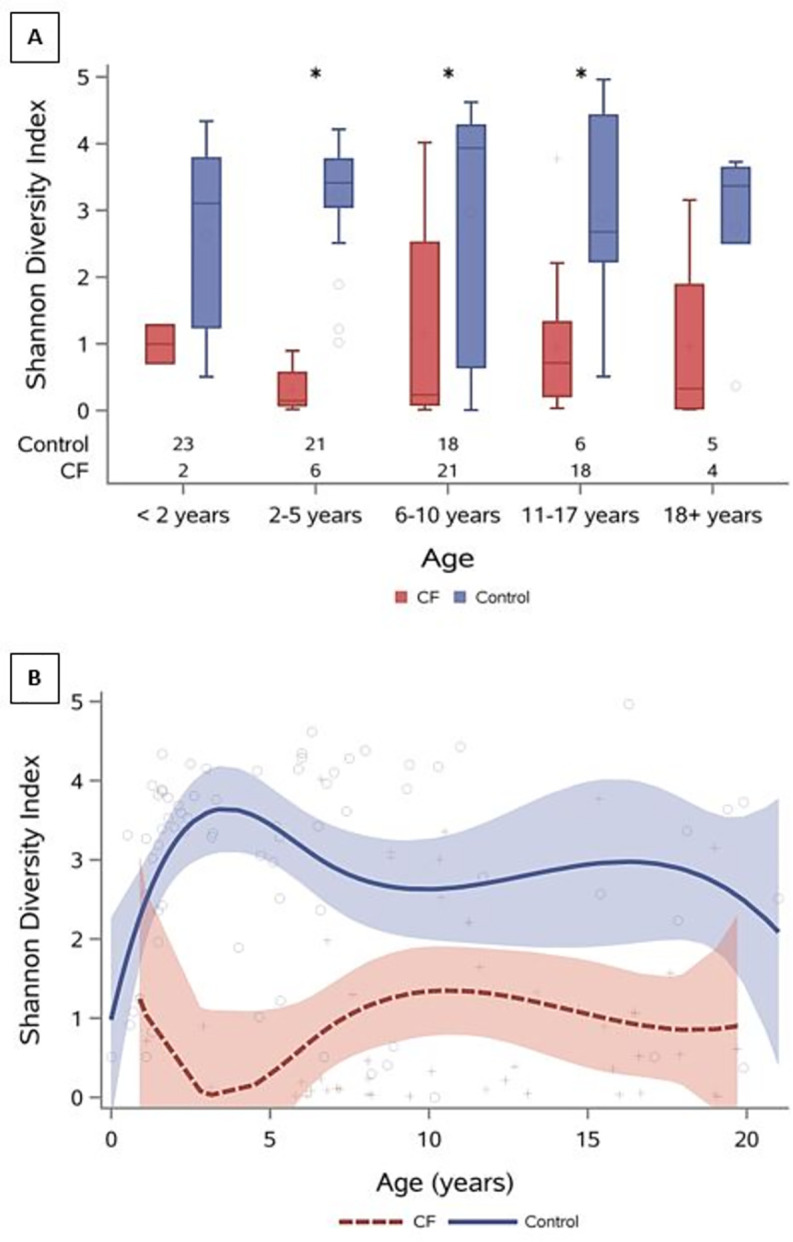
Diversity across age. (A) Bar plots comparing Shannon diversity index between CF and DCs at different ages, sample sizes are displayed along the x-axis and asterisks represent significant differences between CF and DC samples, and (B) Shannon diversity across the age spectrum. On average BALF samples from CF subjects have lower diversity compared to DCs, this is more pronounced starting at 1 year of age.

Shannon diversity index, calculated for BALF samples with sequencing data, was significantly higher in DC samples compared to CF samples (p<0.001, Wilcoxon rank-based test). Diversity was similar between the two groups during infancy and began to diverge after age 2, with DCs showing increased diversity after infancy and CF subjects continuing to exhibit lower diversity across the age spectrum (Fig 2).

### Taxa

One hundred thirty-nine taxa (OTUs) were present in the 124 sequenced samples, which included both species-specific OTUs (i.e. *Streptococcus mitis|oralis|pneumoniae* herein referred to as the *Streptococcus mitis* group) and genus-level OTUs assigned to unspeciated sequences (i.e. *Streptococcus*). The most prevalent taxa are summarized in the supporting information. Highest-ranking taxa, defined as the taxa with the highest relative abundance (RA) in the samples, are summarized in S3 Table, with comparisons also summarized in the supporting information.

**Fig 3:**
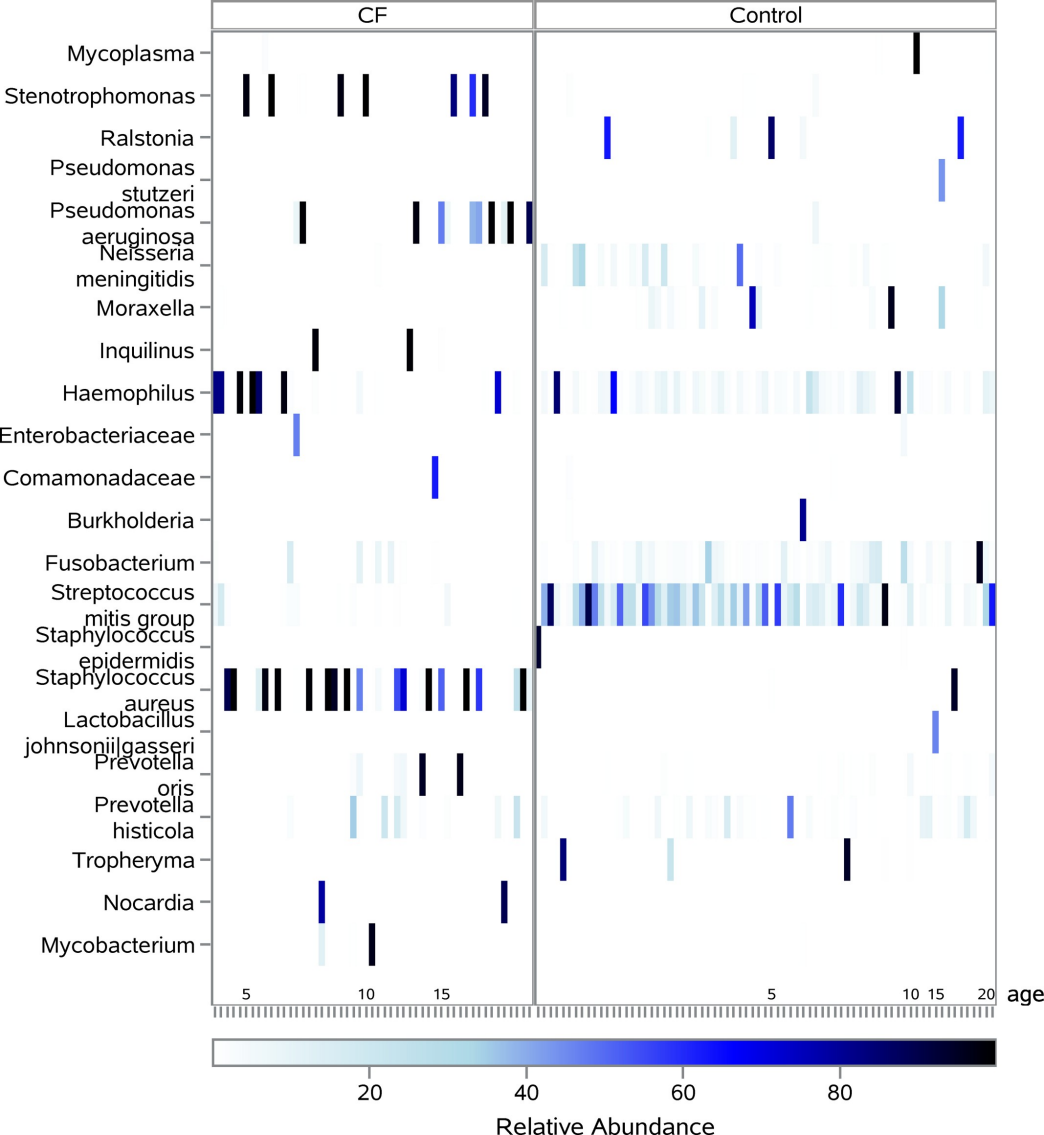
Relative abundance across age. Heatmap of sequencing results for all BALF samples in CF and DC groups with samples arranged in order of increasing age (in years). Taxa that had a relative abundance of over 40% in at least one of the samples are included. Clear distinctions in microbiota across the age spectrum are seen, with any taxa identified in high relative abundance in CF samples being seen in low relative abundance in DC samples (*Staphylococcus aureus*, *Stenotrophomonas*) and any taxa observed in low relative abundance in CF samples seen in high abundance in DC samples (*Streptococcus mitis* group).

Differences in the composition of lower airway communities were observed across the age spectrum. A heat map of the RA for the most prevalent taxa organized by age is shown in Fig 3. Notably, the *Streptococcus mitis* group was present across the age spectrum in DC subjects and was identified only in low abundance in younger CF subjects. *Haemophilus*, which was present across the age spectrum in DCs, was typically only present in CF subjects at younger ages. Conversely, taxa identified in greater abundance in CF subjects included typical CF pathogens, like *Staphylococcus aureus*, *Stenotrophomonas*, and *Pseudomonas aeruginosa*.

Bacterial communities in individual BALF samples grouped by age and cohort are displayed in S4 Fig. CF subjects exhibited decreases in the number of taxa identified, particularly in the 2–5 year old and 6–10 year old age groups. In BALF samples from CF subjects < 2 years of age, there was a greater dominance of *Haemophilus*; however, in the 2–10 year old subjects, we observed the introduction of *Staphylococcus aureus* and *Stenotrophomonas* as a dominant taxa in CF subjects, followed by *Pseudomonas aeruginosa* in older subjects.

### Species level data

Thirty-eight of the total 139 taxa present in the sample set included species-level information. The most prevalent species-level taxa included the *Streptococcus mitis* group (65/124, 52%) and *Prevotella melaninogenica* (N = 54/124,44%). Abundance of the most common nontraditional species-level taxon, *Streptococcus mitis*, and the most common tradition CF-associated species-level taxon, *Staphylococcus aureus*, across the age spectrum are shown in S5 Fig. Additional differences in the prevalent species-level taxa detected in CF and DC groups are summarized in the supporting information.

### Comparison with standard culture

Of the 124 samples successfully sequenced, 100 had a corresponding culture available for comparison (N = 27 CF). S4 Table displays the comparison of 5 traditional CF-related bacterial pathogens (*i*.*e*. *Pseudomonas aeruginosa*, *Staphylococcus aureus*, *Haemophilus*, *Stenotrophomonas*, and *Achromobacter*). Of the samples with CF-related bacterial organisms detected by sequencing, the majority were BALF culture negative, and, of the pathogens identified by sequencing, the RA was higher in culture positive samples (S4 Table).

### Relationship between CF BALF microbiota, airway inflammation, antibiotic use, and clinical characteristics

Sequenced samples were clustered into groups using the Morisita-Horn beta diversity measure (S7 Fig), which is summarized in the supporting information.

Review of antibiotic use in the entire sample set revealed that DC subjects were more frequently exposed to antibiotics, while there was limited number of samples with antibiotic data available (Table 1). While antibiotic use correlated with lower neutrophilic inflammation (p = 0.002, Wilcoxon rank-based test) and lower bacterial burden (p = 0.049, Wilcoxon rank-based test), there were no significant differences in bacterial diversity and lung function in those receiving versus those not receiving antibiotic treatment.

**Fig 4:**
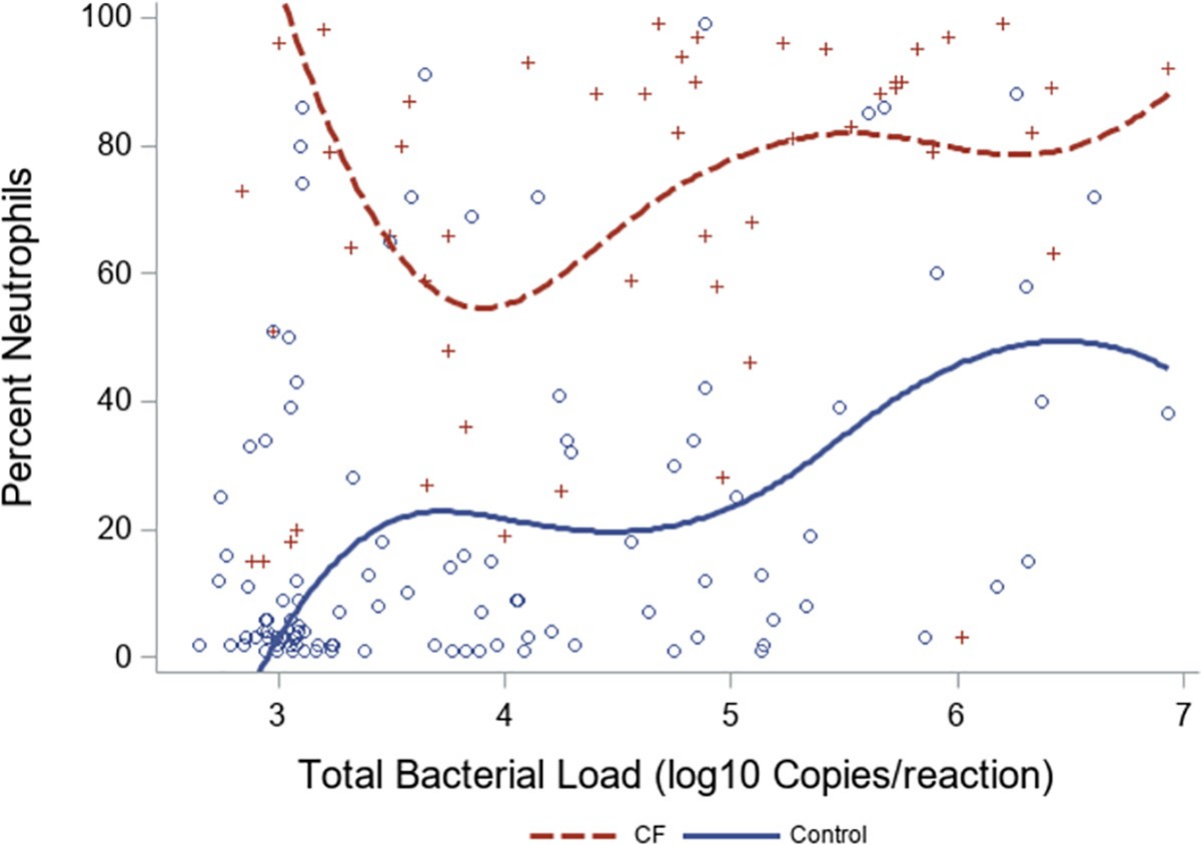
Neutrophilic inflammation. Percent neutrophils across the spectrum of bacterial burden. Notable differences are seen between CF and DC subjects. While CF subjects have heightened percent neutrophils regardless of load, DCs exhibit a stronger linear relationship between percent neutrophils and bacterial burden.

Airway inflammation, as measured by total white blood cell count and percent neutrophils in BALF, of all the samples revealed CF subjects had higher white blood cell counts (p<0.001, Wilcoxon rank-based test) and percent neutrophils (p<0.001, Wilcoxon rank-based test) compared to DCs. Increased inflammation was seen in CF subjects over two years of age (S6 Fig). CF subjects had heightened neutrophilic inflammation even at lower bacterial loads (Fig 4), and while neutrophilic inflammation was positively correlated with TBL in CF samples (p = 0.005, Spearman Correlation) it showed a stronger correlation with TBL in DCs (p = 0.001, Spearman Correlation) (Fig 4). Of the subjects with FEV_1_ percent predicted documented at the time of the flexible bronchoscopy, there were no significant differences between CF and DCs (Table 1).

## Discussion

In this multicenter study, we identified differences in BALF microbiota between people with CF and DC subjects across the age spectrum. Through the use of a comprehensive sample set from subjects ranging from 0 months to 21 years of age with and without CF, we were able to identify differences in TBL, diversity, and airway composition.

Our work confirmed that TBL was higher in CF lower airway samples compared to DCs across the age spectrum [34]. A pronounced difference was observed in early childhood and adolescence, suggesting environmental exposures or developmental changes exhibited during school age could drive microbial changes in the CF airway. Given CF is a disease characterized by chronic airway infection [1, 41, 42], a high bacterial burden is expected; however, such a clear divergence from samples collected from DCs, several of which were immunosuppressed and chronically infected, is noteworthy. In addition to higher TBL, CF subjects had more neutrophilic inflammation compared to DCs. Airway inflammation increased with increasing TBL in DCs but remained high in CF subjects regardless of bacterial burden. This is consistent with previous findings that have shown heightened inflammation in the CF airway even during clinical stability in the absence of infection in both human and animal studies [4, 43–46]. This finding paints a picture of an inflamed lower airway in CF that is not dependent on cellular bacterial burden to trigger neutrophilic influx. In addition, specific communities may be capable of triggering intense neutrophilic inflammation independent of bacterial density, highlighting the need for further investigation.

16S gene-sequencing of BALF confirmed the presence of traditional CF pathogens inhabiting the lower CF airway, including *Stenotrophomonas*, *Staphylococcus aureus*, and *Pseudomonas aeruginos*a [34]. Despite immunosuppression, airway damage and infection, these pathogens were not commonly found in the lower airways of DCs. When comparing the airway composition across the age spectrum, there are additional differences between CF and DC subjects. Accompanying the increased TBL early in life was a decrease in lower airway diversity, which was driven by changes in both evenness, observed upon the introduction of singular dominant taxa, and richness, observed in the depletion of other less abundant taxa. The correlation between decreased diversity and age in CF has confirmed findings in previous studies [24–26, 33, 34, 47]; however, our work identified a clear divergence in diversity between CF and DC subjects starting around age 2 and persisting through adulthood. This difference in diversity was most noticeable between 2–5 years of age, identifying a time when airway infection, inflammation and mucus obstruction may be taking hold within the CF airway. Bacterial communities of the lower CF airway are distinctly different from those without CF, but the CF airway also remains unique in its ability to house intense neutrophilic inflammation and high TBL, setting the stage for chronic lung disease and bronchiectasis. Such differences between subjects with CF and DC subjects, who had a variety of indications and underlying diagnoses, is noteworthy. While smaller sample numbers preclude making strong inferences, this 2–5 year old age range may represent a window of opportunity for intervention with additional study of the bacterial communities and how they interact with the airway environment.

In the largest analysis of lower airways samples from a heterogeneous group of DCs, increased diversity appeared to be independent of indication for bronchoscopy or primary diagnoses. While additional investigation is needed, this diverse airway composition may be representative of healthy pediatric airway ecology, which has been reported when comparing the airway microbiota of healthy adults to that of other disease types [48–50]. A similar divergence in diversity between healthy infants and infants with CF has previously been shown in the gut microbiome [51]. Further exploration of microbial trends in DC samples may help us better characterize the normal airway ecology to understand what is normal how and when CF subjects start to diverge from typical community succession.

Our study provided a glimpse into specific bacterial communities present in the lower airways across the pediatric population. The presence and eventual dominance of the lower airway community of traditional CF pathogens later in life coincided with the depletion of nontraditional CF taxa. In the earliest CF samples, in addition to seeing *Haemophilus* dominance, we saw a higher relative abundance of the nontraditional *Streptococcus mitis* group which is consistent with the previous identification of similar nontraditional CF taxa in the infant CF airway [30]. After the age of 2; however, *Stenotrophomonas* and *Staphylococcus aureus* emerged as dominant taxa in the airway of CF patients, which accompanied a decrease in the relative abundance of the *Streptococcus mitis* group, an increase in TBL, and a drop in diversity. The role of anaerobic communities in the lower CF airway (i.e. non-traditional CF taxa) has been debated by us and others [30, 52–56]. In vitro studies have shown that oral mucin-degrading anaerobes can provide a nutrient source for the growth of traditional CF pathogens [57]. So, while our work suggests that such traditional CF pathogens like *Stenotrophomonas* and *S*. *aureus* may serve as significant drivers of microbial changes in the CF airway, the role of nontraditional bacterial communities in the creation of an airway environment conducive to traditional pathogens like *P*. *aeruginosa* is also suggested and warrants additional study.

Review of the 16S detection and culture results for the five traditional CF pathogens revealed a general agreement between the two methods. Sequencing identified pathogens in culture negative samples more often than culture did in sequence negative samples, indicating potentially greater sensitivity in sequencing. Of the samples with pathogens identified through 16S rRNA gene sequencing, those that were culture positive often had higher relative abundances, indicating a correlation between the abundance of a particular pathogen and whether it is detected in culture. Comparisons of FEV_1_ percent predicted between CF and DC samples revealed no notable differences. While progressive lung function decline is expected to be more dramatic in the CF cohort, the lack of significant difference could be attributed to the other underlying diagnoses and indications for bronchoscopy in our DC cohort. Additionally, while antibiotics data was limited, DC subjects were more often taking antibiotic medications at the time of sample collection. This could be expected when considering the underlying diagnoses of our DC subjects and the fact that CF subjects may have been started on antibiotics after bronchoscopy. Neutrophilic inflammation and bacterial burden vary by antibiotic exposure, indicating that antibiotic exposure could be affecting the differences observed in the two disease groups.

Our study is not without limitations. Given the multicenter nature of this study, while our samples were processed the same way to allow for 16S sequencing, they were not collected in a uniform fashion. While only 9 neat samples were included with the majority pellet samples used in our analysis, the impact of the level of processing on measured total bacterial load has not been investigated. Similarly, subjectivity in diagnoses, inconsistency in reporting of culture results, and the varied availability of associated clinical data at different institutions may have impacted our analyses. We included samples from subjects across the age spectrum. While we had a limited number of samples from the CF subjects under 5 years of age, where we saw changes in diversity and bacterial burden, our findings are consistent with previous studies [24–26, 33, 34, 47]. More importantly, BALF was collected during clinically indicated bronchoscopies; therefore, the communities identified represent a state that may differ from baseline stability. Flexible bronchoscopy for research purposes in children is not routinely done in the United States, limiting the procurement of such specimens. Similarly, because our DC samples underwent bronchoscopies for a variety of indications, interpretation of the results is limited by the heterogeneity of underlying diseases.

In this multicenter study of BALF samples, we found distinctions between the airway microbiomes of CF and DC subjects in a pediatric population that can inform future study. By using 16S, we characterized complex polymicrobial communities in the lower airway from infancy up to young adulthood in people with and without CF. A more in-depth evaluation of the airway microbiome and how it incites inflammation early in life may provide valuable insight into the development of CF lung disease with the potential for interventions to mitigate disease progression.

## Supporting information

S1 Fig Background comparison of relative abundance of 12 most abundant taxa. Box plots of the relative abundance distribution of the 12 most abundant taxa in the sample set with *Acinetobacter* being the twelfth most abundant and *Staphylococcus* being the most abundant. (A) represents CF samples, (B) represents DC samples, and (C) represents negative controls. Boxes show 25–75th interquartile range (IQR) with whiskers showing 1.5 times the IQR. Median is indicated by a solid line in the box. Outliers are shown as individual data points. The negative controls are notably different from the CF and DC samples indicating little contribution of background reagents to the airway microbial compositions.
(TIF)

S2 Fig Ordination analysis of negative control samples. PCoA plot of background samples compared to BALF samples (C). The compositions of the negative control samples were generally distinct from the BALF samples indicating that the background contributes minimally to the composition in the BALF samples.
(TIF)

S3 Fig TBL comparison. Box plots of TBL based on availability of sequencing data (left are samples that failed sequencing and right are samples that were successfully sequenced). Boxes show 25–75th interquartile range (IQR) with whiskers showing 1.5 times the IQR. Median is indicated by a solid line in the box. Outliers are shown as individual data points. Sample sizes are displayed along the x-axis and asterisks represent significant differences between all samples with sequencing data available and all samples without sequencing data available and CF and DC samples with sequencing data available. TBL was higher in samples with successful amplification and sequencing when compared to those that failed sequencing, and within the samples with successful sequencing TBL was higher in CF samples compared to DCs. Therefore, higher load corresponded with a higher likelihood of successful amplification and sequencing and CF subjects generally had higher bacterial load than DCs.
(TIF)

S4 Fig Taxa by age group. Sequencing results of CF and DC subjects organized by age. In CF subjects, we see more dominance of traditional CF pathogens, including *Hemophilus* (<2 years), *Staphylococcus aureus* (2–5 years through 18 years and older), and *Pseudomonas aeruginosa* (18 years and older). In DCs, we see a high prevalence of the *Streptococcus mitis* group.
(TIF)

S5 Fig *Staphylococcus aureus* and *Streptococcus mitis* group. Median relative abundance of *Staphylococcus aureus* (A) and the *Streptococcus mitis* group (B) across the age spectrum. *Staphylococcus aureus* is seen in higher abundance across the age spectrum in CF subjects when compared to DCs. The abundance of the *Streptococcus mitis* group axon was found to be generally higher in younger subjects and diminished overtime, with an earlier drop observed in CF subjects over the age of 2. Whiskers extend to the 25th and 75th percentiles.
(TIF)

S6 Fig Inflammation across age. Box plots of white blood cell counts (A) and neutrophil percentages (B) in CF and DC subjects across the age spectrum. Sample sizes are displayed along the x-axis and asterisks represent significant differences between CF and DC subjects. CF subjects exhibit higher measures of these inflammatory markers when compared to DCs.
(TIF)

S7 Fig Clustering analysis. Clustering analysis of samples based on CF status, primary diagnoses, markers of inflammation, weight, age, bacterial load, diversity, and relative abundance of common taxa using the Morisita-Horn beta diversity measure.
(TIF)

S8 Fig Repeat samples. Total bacterial load (A), Shannon diversity (B) and bacterial composition (C) across the age spectrum for repeat samples from the same subjects.
(TIF)

S1 Table Indications and primary diagnoses. Indications and Primary diagnoses of DC subjects sorted by frequency. Indication/primary diagnosis categories were not mutually exclusive with subjects falling into as many as 3 different indications.
(TIF)

S2 Table Sample characteristics by sequencing. Data are presented as n, median (range) or n (%), unless otherwise states.CF: Cystic fibrosis; FEV1: Forced expiratory volume in 1 s; BALF: Bronchoalveolar lavage fluid; MSSA: Methicillin-susceptible Staphylococcus aureus; MRSA.
(TIF)

S3 Table Dominant taxa. Dominant taxa identified by sequencing in bronchoalveolar lavage fluid (BALF) samples from DC and cystic fibrosis (CF) subjects *Taxa associated as typical CF pathogens. Traditionally CF pathogens appear to dominate CF samples more often than DCs.
(TIF)

S4 Table Comparison to culture. Comparison of BALF culture and BALF sequencing for the identification of five common CF bacterial pathogens. + = bacteria identified either by culture or 16S, - = Not identified, *Some relative abundances are not provided as some taxa were not identified by 16S and therefore relative abundances could not be calculated.
(TIF)

S1 File (DOCX)

## References

[1] Gibson RL, Burns JL, Ramsey BW. Pathophysiology and management of pulmonary infections in cystic fibrosis. Am J Respir Crit Care Med. 2003 Oct 15;168(8):918–51. doi: 10.1164/rccm.200304-505SO 14555458

[2] Pezzulo AA, Tang XX, Hoegger MJ, Abou Alaiwa MH, Ramachandran S, Moninger TO, et al. Reduced airway surface pH impairs bacterial killing in the porcine cystic fibrosis lung [Internet]. Vol. 487, Nature. 2012. p. 109–13. Available from: doi: 10.1038/nature11130 22763554 PMC3390761

[3] LiPuma JJ. Assessing Airway Microbiota in Cystic Fibrosis: What More Should Be Done? J Clin Microbiol. 2015 Jul;53(7):2006–7. doi: 10.1128/JCM.01218-15 25972422 PMC4473192

[4] Keiser NW, Birket SE, Evans IA, Tyler SR, Crooke AK, Sun X, et al. Defective Innate Immunity and Hyperinflammation in Newborn Cystic Fibrosis Transmembrane Conductance Regulator–Knockout Ferret Lungs [Internet]. Vol. 52, American Journal of Respiratory Cell and Molecular Biology. 2015. p. 683–94. Available from: doi: 10.1165/rcmb.2014-0250OC 25317669 PMC4491130

[5] Burns JL, Rolain J-M. Culture-based diagnostic microbiology in cystic fibrosis: can we simplify the complexity? J Cyst Fibros. 2014 Jan;13(1):1–9. doi: 10.1016/j.jcf.2013.09.004 24094376

[6] Razvi S, Quittell L, Sewall A, Quinton H, Marshall B, Saiman L. Respiratory Microbiology of Patients With Cystic Fibrosis in the United States, 1995 to 2005 [Internet]. Vol. 136, Chest. 2009. p. 1554–60. Available from: doi: 10.1378/chest.09-0132 19505987

[7] Shreve MR, Butler S, Kaplowitz HJ, Rabin HR, Stokes D, Light M, et al. Impact of microbiology practice on cumulative prevalence of respiratory tract bacteria in patients with cystic fibrosis. J Clin Microbiol. 1999 Mar;37(3):753–7. doi: 10.1128/JCM.37.3.753-757.1999 9986845 PMC84543

[8] Goss CH, Otto K, Aitken ML, Rubenfeld GD. DetectingStenotrophomonas maltophiliaDoes Not Reduce Survival of Patients with Cystic Fibrosis [Internet]. Vol. 166, American Journal of Respiratory and Critical Care Medicine. 2002. p. 356–61. Available from: doi: 10.1164/rccm.2109078 12153970

[9] Goss CH, Mayer-Hamblett N, Aitken ML, Rubenfeld GD, Ramsey BW. Association between Stenotrophomonas maltophilia and lung function in cystic fibrosis. Thorax. 2004 Nov;59(11):955–9. doi: 10.1136/thx.2003.017707 15516471 PMC1746887

[10] Sibley CD, Rabin H, Surette MG. Cystic fibrosis: a polymicrobial infectious disease [Internet]. Vol. 1, Future Microbiology. 2006. p. 53–61. Available from: doi: 10.2217/17460913.1.1.53 17661685

[11] Hoen AG, Li J, Moulton LA, O’Toole GA, Housman ML, Koestler DC, et al. Associations between Gut Microbial Colonization in Early Life and Respiratory Outcomes in Cystic Fibrosis. J Pediatr. 2015 Jul;167(1):138–47.e1–3. doi: 10.1016/j.jpeds.2015.02.049 25818499 PMC4674690

[12] Prevaes SMPJ, de Winter-de Groot KM, Janssens HM, de Steenhuijsen Piters WAA, Tramper-Stranders GA, Wyllie AL, et al. Development of the Nasopharyngeal Microbiota in Infants with Cystic Fibrosis. Am J Respir Crit Care Med. 2016 Mar 1;193(5):504–15. doi: 10.1164/rccm.201509-1759OC 26492486

[13] Coenye T, Goris J, Spilker T, Vandamme P, LiPuma JJ. Characterization of unusual bacteria isolated from respiratory secretions of cystic fibrosis patients and description of Inquilinus limosus gen. nov., sp. nov. J Clin Microbiol. 2002 Jun;40(6):2062–9. doi: 10.1128/JCM.40.6.2062-2069.2002 12037065 PMC130740

[14] Willner D, Haynes MR, Furlan M, Schmieder R, Lim YW, Rainey PB, et al. Spatial distribution of microbial communities in the cystic fibrosis lung. ISME J. 2012 Feb;6(2):471–4. doi: 10.1038/ismej.2011.104 21796216 PMC3260497

[15] Lim YW, Evangelista JS 3rd, Schmieder R, Bailey B, Haynes M, Furlan M, et al. Clinical insights from metagenomic analysis of sputum samples from patients with cystic fibrosis. J Clin Microbiol. 2014 Feb;52(2):425–37. doi: 10.1128/JCM.02204-13 24478471 PMC3911355

[16] Bittar F, Richet H, Dubus J-C, Reynaud-Gaubert M, Stremler N, Sarles J, et al. Molecular detection of multiple emerging pathogens in sputa from cystic fibrosis patients. PLoS One. 2008 Aug 6;3(8):e2908. 10.1371/journal.pone.0002908 PMC2483419 18682840

[17] Rogers GB, Carroll MP, Serisier DJ, Hockey PM, Jones G, Bruce KD. Characterization of bacterial community diversity in cystic fibrosis lung infections by use of 16s ribosomal DNA terminal restriction fragment length polymorphism profiling. J Clin Microbiol. 2004 Nov;42(11):5176–83. doi: 10.1128/JCM.42.11.5176-5183.2004 15528712 PMC525137

[18] Feigelman R, Kahlert CR, Baty F, Rassouli F, Kleiner RL, Kohler P, et al. Sputum DNA sequencing in cystic fibrosis: non-invasive access to the lung microbiome and to pathogen details. Microbiome. 2017 Feb 10;5(1):20. doi: 10.1186/s40168-017-0234-1 28187782 PMC5303297

[19] Jorth P, Ehsan Z, Rezayat A, Caldwell E, Pope C, Brewington JJ, et al. Direct Lung Sampling Indicates That Established Pathogens Dominate Early Infections in Children with Cystic Fibrosis. Cell Rep. 2019 Apr 23;27(4):1190–204.e3. doi: 10.1016/j.celrep.2019.03.086 31018133 PMC6668708

[20] Tracy M, Cogen J, Hoffman LR. The pediatric microbiome and the lung. Curr Opin Pediatr. 2015 Jun;27(3):348–55. doi: 10.1097/MOP.0000000000000212 25888147 PMC4443818

[21] Hisert KB, Heltshe SL, Pope C, Jorth P, Wu X, Edwards RM, et al. Restoring Cystic Fibrosis Transmembrane Conductance Regulator Function Reduces Airway Bacteria and Inflammation in People with Cystic Fibrosis and Chronic Lung Infections. Am J Respir Crit Care Med. 2017 Jun 15;195(12):1617–28. doi: 10.1164/rccm.201609-1954OC 28222269 PMC5476912

[22] Rowe SM, Heltshe SL, Gonska T, Donaldson SH, Borowitz D, Gelfond D, et al. Clinical mechanism of the cystic fibrosis transmembrane conductance regulator potentiator ivacaftor in G551D-mediated cystic fibrosis. Am J Respir Crit Care Med. 2014 Jul 15;190(2):175–84. doi: 10.1164/rccm.201404-0703OC 24927234 PMC4226057

[23] Bernarde C, Keravec M, Mounier J, Gouriou S, Rault G, Férec C, et al. Impact of the CFTR-potentiator ivacaftor on airway microbiota in cystic fibrosis patients carrying a G551D mutation. PLoS One. 2015 Apr 8;10(4):e0124124. doi: 10.1371/journal.pone.0124124 25853698 PMC4390299

[24] Klepac-Ceraj V, Lemon KP, Martin TR, Allgaier M, Kembel SW, Knapp AA, et al. Relationship between cystic fibrosis respiratory tract bacterial communities and age, genotype, antibiotics and Pseudomonas aeruginosa. Environ Microbiol. 2010 May;12(5):1293–303. doi: 10.1111/j.1462-2920.2010.02173.x 20192960

[25] Paganin P, Fiscarelli EV, Tuccio V, Chiancianesi M, Bacci G, Morelli P, et al. Changes in cystic fibrosis airway microbial community associated with a severe decline in lung function. PLoS One. 2015 Apr 21;10(4):e0124348. doi: 10.1371/journal.pone.0124348 25898134 PMC4405530

[26] Coburn B, Wang PW, Diaz Caballero J, Clark ST, Brahma V, Donaldson S, et al. Lung microbiota across age and disease stage in cystic fibrosis. Sci Rep. 2015 May 14;5:10241. doi: 10.1038/srep10241 25974282 PMC4431465

[27] Ahmed B, Cox MJ, Cuthbertson L, James P, Cookson WOC, Davies JC, et al. Longitudinal development of the airway microbiota in infants with cystic fibrosis. Sci Rep. 2019 Mar 26;9(1):5143. doi: 10.1038/s41598-019-41597-0 30914718 PMC6435666

[28] Goddard AF, Staudinger BJ, Dowd SE, Joshi-Datar A, Wolcott RD, Aitken ML, et al. Direct sampling of cystic fibrosis lungs indicates that DNA-based analyses of upper-airway specimens can misrepresent lung microbiota. Proc Natl Acad Sci U S A. 2012 Aug 21;109(34):13769–74. doi: 10.1073/pnas.1107435109 22872870 PMC3427132

[29] Kirst ME, Baker D, Li E, Abu-Hasan M, Wang GP. Upper versus lower airway microbiome and metagenome in children with cystic fibrosis and their correlation with lung inflammation. PLoS One. 2019 Sep 19;14(9):e0222323. doi: 10.1371/journal.pone.0222323 31536536 PMC6752789

[30] Laguna TA, Wagner BD, Williams CB, Stevens MJ, Robertson CE, Welchlin CW, et al. Airway Microbiota in Bronchoalveolar Lavage Fluid from Clinically Well Infants with Cystic Fibrosis [Internet]. Vol. 11, PLOS ONE. 2016. p. e0167649. Available from: doi: 10.1371/journal.pone.0167649 27930727 PMC5145204

[31] Frayman KB, Wylie KM, Armstrong DS, Carzino R, Davis SD, Ferkol TW, et al. Differences in the lower airway microbiota of infants with and without cystic fibrosis. J Cyst Fibros. 2019 Sep;18(5):646–52. doi: 10.1016/j.jcf.2018.12.003 30580994 PMC6586525

[32] Harris JK, De Groote MA, Sagel SD, Zemanick ET, Kapsner R, Penvari C, et al. Molecular identification of bacteria in bronchoalveolar lavage fluid from children with cystic fibrosis. Proc Natl Acad Sci U S A. 2007 Dec 18;104(51):20529–33. doi: 10.1073/pnas.0709804104 18077362 PMC2154465

[33] Renwick J, McNally P, John B, DeSantis T, Linnane B, Murphy P, et al. The microbial community of the cystic fibrosis airway is disrupted in early life. PLoS One. 2014 Dec 19;9(12):e109798. doi: 10.1371/journal.pone.0109798 25526264 PMC4272276

[34] Zemanick ET, Wagner BD, Robertson CE, Ahrens RC, Chmiel JF, Clancy JP, et al. Airway microbiota across age and disease spectrum in cystic fibrosis. Eur Respir J [Internet]. 2017 Nov;50(5). Available from: doi: 10.1183/13993003.00832-2017 29146601 PMC5935257

[35] O’Connor JB, Wright JC, Wagner BD, Harris JK, Laguna TA. Divergence of the bacterial communities in the lower airways of cystic fibrosis patients in early childhood. In: D71 CYSTIC FIBROSIS AND DISORDERS OF MUCOCILIARY APPARATUS [Internet]. American Thoracic Society; 2020. Available from: https://www.atsjournals.org/doi/10.1164/ajrccm-conference.2020.201.1_MeetingAbstracts.A7456

[36] O’Connor JB, Wright JC, Wagner BD, Harris JK, Laguna TA. Divergence of the Bacterial Communities in the Lower Airways of Cystic Fibrosis Patients in Early Childhood. The 34 Annual North American Cystic Fibrosis Conference, October 7–23, 2020. Pediatr Pulmonol 2020;55 Suppl 2:S179–S180

[37] Nadkarni MA, Martin FE, Jacques NA, Hunter N. Determination of bacterial load by real-time PCR using a broad-range (universal) probe and primers set. Microbiology. 2002 Jan;148(Pt 1):257–66. doi: 10.1099/00221287-148-1-257 11782518

[38] Zemanick ET, Wagner BD, Sagel SD, Stevens MJ, Accurso FJ, Harris JK. Reliability of quantitative real-time PCR for bacterial detection in cystic fibrosis airway specimens. PLoS One. 2010 Nov 30;5(11):e15101. doi: 10.1371/journal.pone.0015101 21152087 PMC2994853

[39] Hara N, Alkanani AK, Ir D, Robertson CE, Wagner BD, Frank DN, et al. Prevention of virus-induced type 1 diabetes with antibiotic therapy. J Immunol. 2012 Oct 15;189(8):3805–14. doi: 10.4049/jimmunol.1201257 22988033

[40] Markle JGM, Frank DN, Mortin-Toth S, Robertson CE, Feazel LM, Rolle-Kampczyk U, et al. Sex differences in the gut microbiome drive hormone-dependent regulation of autoimmunity. Science. 2013 Mar 1;339(6123):1084–8. doi: 10.1126/science.1233521 23328391

[41] Lyczak JB, Cannon CL, Pier GB. Lung infections associated with cystic fibrosis. Clin Microbiol Rev. 2002 Apr;15(2):194–222. doi: 10.1128/CMR.15.2.194-222.2002 11932230 PMC118069

[42] Govan JRW, Nelson JW. Microbiology of lung infection in cystic fibrosis [Internet]. Vol. 48, British Medical Bulletin. 1992. p. 912–30. Available from: doi: 10.1093/oxfordjournals.bmb.a072585 1281036

[43] Sagel SD, Accurso FJ. Monitoring Inflammation in CF: Cytokines [Internet]. Vol. 23, Clinical Reviews in Allergy & Immunology. 2002. p. 041–58. Available from: doi: 10.1385/CRIAI:23:1:041 12162105

[44] Hendry J, Elborn JS, Nixon L, Shale DJ, Webb AK. Cystic fibrosis: inflammatory response to infection with Burkholderia cepacia and Pseudomonas aeruginosa. Eur Respir J. 1999 Aug;14(2):435–8. doi: 10.1034/j.1399-3003.1999.14b32.x 10515426

[45] Pillarisetti N, Williamson E, Linnane B, Skoric B, Robertson CF, Robinson P, et al. Infection, inflammation, and lung function decline in infants with cystic fibrosis. Am J Respir Crit Care Med. 2011 Jul 1;184(1):75–81. doi: 10.1164/rccm.201011-1892OC 21493738

[46] Rosen BH, Idil Apak Evans T, Moll SR, Gray JS, Liang B, Sun X, et al. Infection Is Not Required for Mucoinflammatory Lung Disease in CFTR-Knockout Ferrets [Internet]. Vol. 197, American Journal of Respiratory and Critical Care Medicine. 2018. p. 1308–18. Available from: doi: 10.1164/rccm.201708-1616OC 29327941 PMC5955060

[47] Guss AM, Roeselers G, Newton ILG, Young CR, Klepac-Ceraj V, Lory S, et al. Phylogenetic and metabolic diversity of bacteria associated with cystic fibrosis. ISME J. 2011 Jan;5(1):20–9. doi: 10.1038/ismej.2010.88 20631810 PMC3105664

[48] Botero LE, Delgado-Serrano L, Cepeda ML, Bustos JR, Anzola JM, Del Portillo P, et al. Respiratory tract clinical sample selection for microbiota analysis in patients with pulmonary tuberculosis. Microbiome. 2014;2: 29. doi: 10.1186/2049-2618-2-29 25225609 PMC4164332

[49] Wang K, Huang Y, Zhang Z, Liao J, Ding Y, Fang X, et al. A Preliminary Study of Microbiota Diversity in Saliva and Bronchoalveolar Lavage Fluid from Patients with Primary Bronchogenic Carcinoma. Med Sci Monit. 2019;25: 2819–2834. doi: 10.12659/MSM.915332 30994108 PMC6482867

[50] Philley JV, Kannan A, Olusola P, McGaha P, Singh KP, Samten B, et al. Microbiome Diversity in Sputum of Nontuberculous Mycobacteria Infected Women with a History of Breast Cancer. Cell Physiol Biochem. 2019;52: 263–279. doi: 10.33594/000000020 30816674

[51] Hayden HS, Eng A, Pope CE, Brittnacher MJ, Vo AT, Weiss EJ, et al. Fecal dysbiosis in infants with cystic fibrosis is associated with early linear growth failure. Nat Med. 2020 Feb;26(2):215–21. doi: 10.1038/s41591-019-0714-x 31959989 PMC7018602

[52] Zemanick ET, Wagner BD, Robertson CE, Stevens MJ, Szefler SJ, Accurso FJ, et al. Assessment of airway microbiota and inflammation in cystic fibrosis using multiple sampling methods. Ann Am Thorac Soc. 2015 Feb;12(2):221–9. doi: 10.1513/AnnalsATS.201407-310OC 25474078 PMC4342834

[53] Zemanick ET, Harris JK, Wagner BD, Robertson CE, Sagel SD, Stevens MJ, et al. Inflammation and airway microbiota during cystic fibrosis pulmonary exacerbations. PLoS One. 2013 Apr 30;8(4):e62917. doi: 10.1371/journal.pone.0062917 23646159 PMC3639911

[54] Mirković B, Murray MA, Lavelle GM, Molloy K, Azim AA, Gunaratnam C, et al. The Role of Short-Chain Fatty Acids, Produced by Anaerobic Bacteria, in the Cystic Fibrosis Airway. Am J Respir Crit Care Med. 2015 Dec 1;192(11):1314–24. doi: 10.1164/rccm.201505-0943OC 26266556 PMC4731701

[55] Bradshaw DJ, Homer KA, Marsh PD, Beighton D. Metabolic cooperation in oral microbial communities during growth on mucin. Microbiology. 1994 Dec;140 (Pt 12):3407–12. doi: 10.1099/13500872-140-12-3407 7881558

[56] Wright DP, Rosendale DI, Robertson AM. Prevotella enzymes involved in mucin oligosaccharide degradation and evidence for a small operon of genes expressed during growth on mucin. FEMS Microbiol Lett. 2000 Sep 1;190(1):73–9. doi: 10.1111/j.1574-6968.2000.tb09265.x 10981693

[57] Flynn JM, Niccum D, Dunitz JM, Hunter RC. Evidence and Role for Bacterial Mucin Degradation in Cystic Fibrosis Airway Disease. PLoS Pathog. 2016 Aug;12(8):e1005846. doi: 10.1371/journal.ppat.1005846 27548479 PMC4993466

