# Supplementary figures and images for: Divergence of bacterial communities in the lower airways of CF patients in early childhood
Source: PLoS One. 2021 Oct 6;16(10):e0257838. doi: 10.1371/journal.pone.0257838 (PMC8494354; doi:10.1371/journal.pone.0257838)

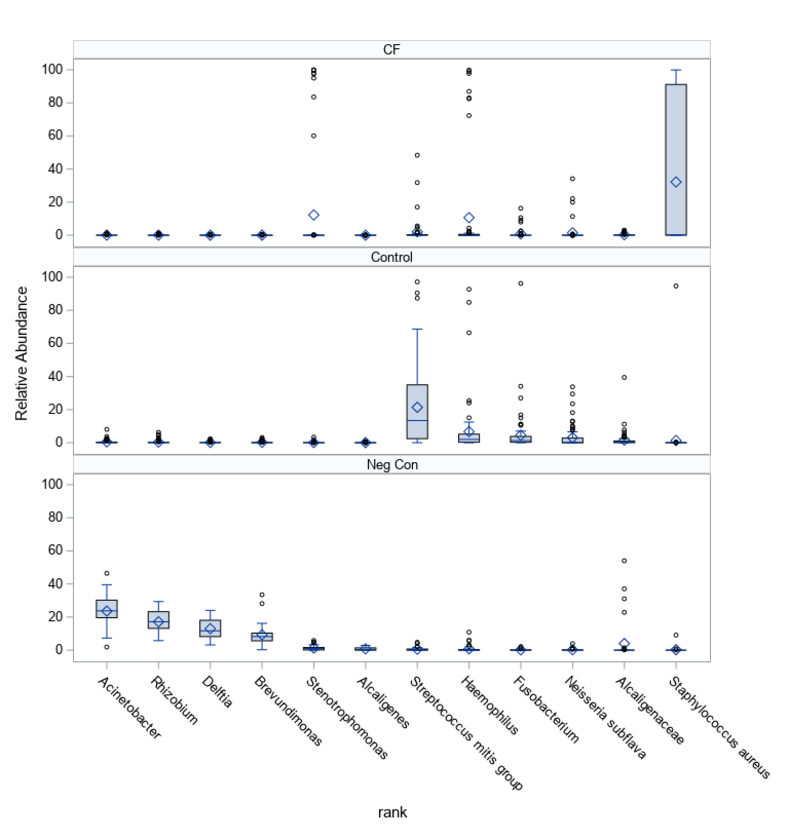

Supplement: S1 Fig — Box plots of the relative abundance distribution of the 12 most abundant taxa in the sample set with Acinetobacter being the twelfth most abundant and Staphylococcus being the most abundant. (A) represents CF samples, (B) represents DC samples, and (C) represents negative controls. Boxes show 25–75th interquartile range (IQR) with whiskers showing 1.5 times the IQR. Median is indicated by a solid line in the box. Outliers are shown as individual data points. The negative controls are notably different from the CF and DC samples indicating little contribution of background reagents to the airway microbial compositions. (TIF) [file pone.0257838.s001.tif]

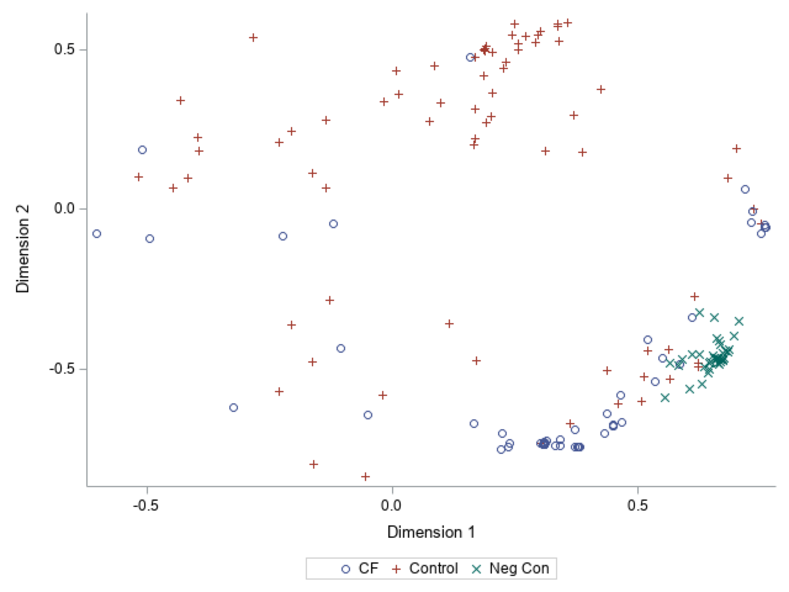

Supplement: S2 Fig — PCoA plot of background samples compared to BALF samples (C). The compositions of the negative control samples were generally distinct from the BALF samples indicating that the background contributes minimally to the composition in the BALF samples. (TIF) [file pone.0257838.s002.tif]

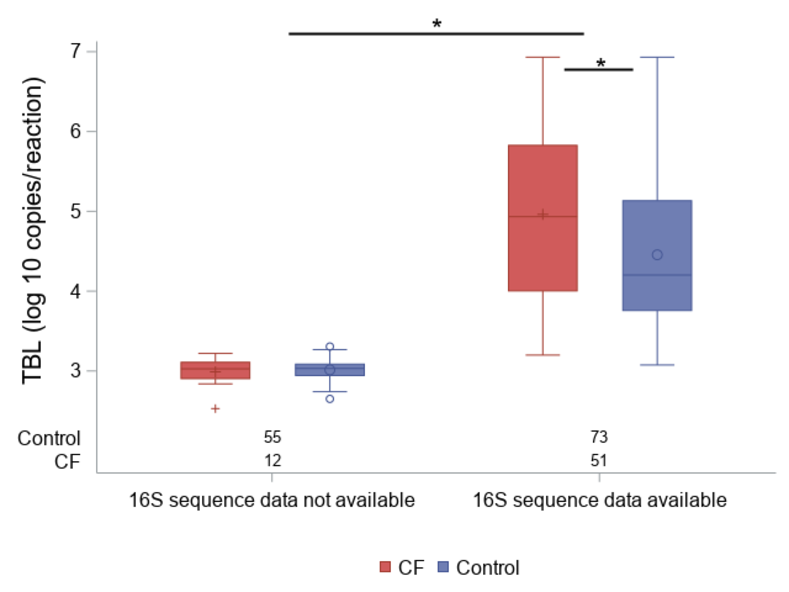

Supplement: S3 Fig — Box plots of TBL based on availability of sequencing data (left are samples that failed sequencing and right are samples that were successfully sequenced). Boxes show 25–75th interquartile range (IQR) with whiskers showing 1.5 times the IQR. Median is indicated by a solid line in the box. Outliers are shown as individual data points. Sample sizes are displayed along the x-axis and asterisks represent significant differences between all samples with sequencing data available and all samples without sequencing data available and CF and DC samples with sequencing data available. TBL was higher in samples with successful amplification and sequencing when compared to those that failed sequencing, and within the samples with successful sequencing TBL was higher in CF samples compared to DCs. Therefore, higher load corresponded with a higher likelihood of successful amplification and sequencing and CF subjects generally had higher bacterial load than DCs. (TIF) [file pone.0257838.s003.tif]

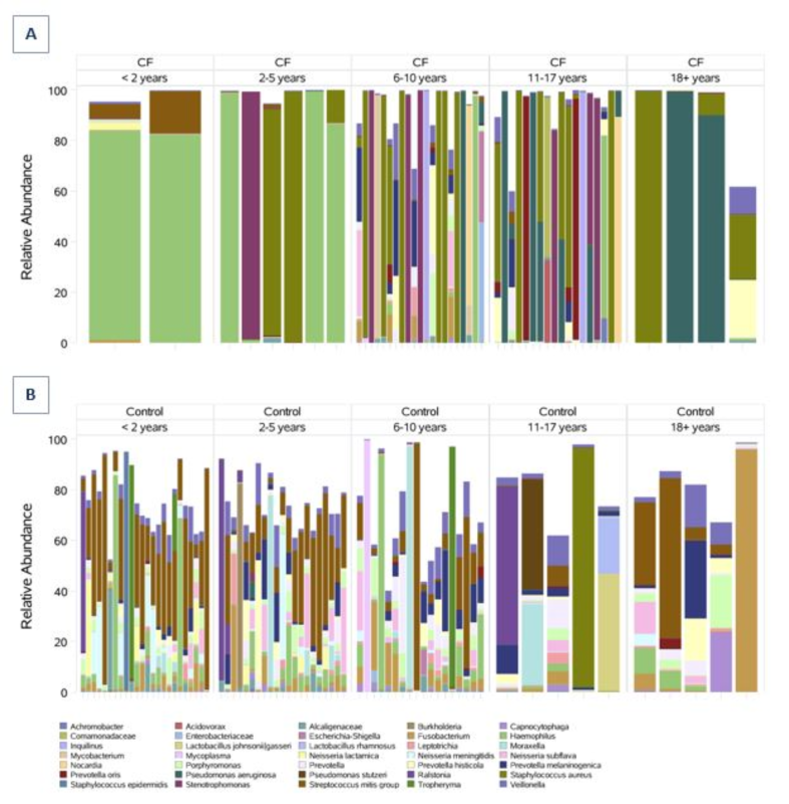

Supplement: S4 Fig — Sequencing results of CF and DC subjects organized by age. In CF subjects, we see more dominance of traditional CF pathogens, including Hemophilus (<2 years), Staphylococcus aureus (2–5 years through 18 years and older), and Pseudomonas aeruginosa (18 years and older). In DCs, we see a high prevalence of the Streptococcus mitis group. (TIF) [file pone.0257838.s004.tif]

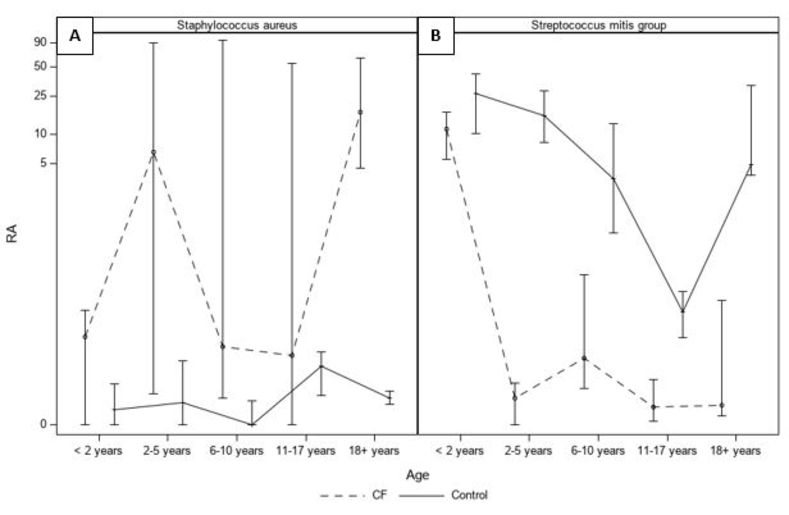

Supplement: S5 Fig — Median relative abundance of Staphylococcus aureus (A) and the Streptococcus mitis group (B) across the age spectrum. Staphylococcus aureus is seen in higher abundance across the age spectrum in CF subjects when compared to DCs. The abundance of the Streptococcus mitis group axon was found to be generally higher in younger subjects and diminished overtime, with an earlier drop observed in CF subjects over the age of 2. Whiskers extend to the 25th and 75th percentiles. (TIF) [file pone.0257838.s005.tif]

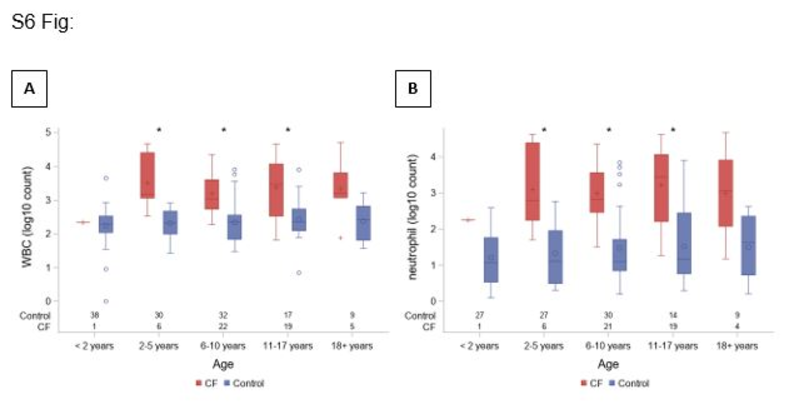

Supplement: S6 Fig — Box plots of white blood cell counts (A) and neutrophil percentages (B) in CF and DC subjects across the age spectrum. Sample sizes are displayed along the x-axis and asterisks represent significant differences between CF and DC subjects. CF subjects exhibit higher measures of these inflammatory markers when compared to DCs. (TIF) [file pone.0257838.s006.tif]

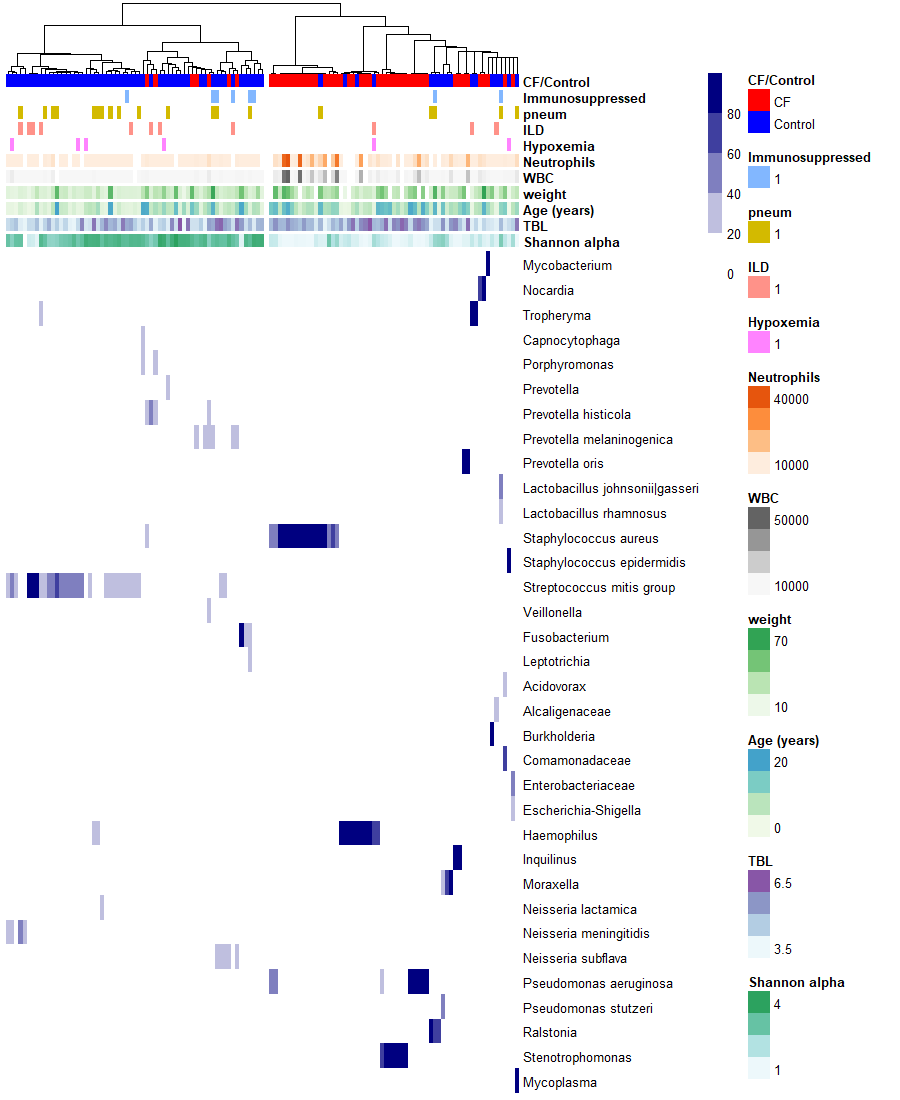

Supplement: S7 Fig — Clustering analysis of samples based on CF status, primary diagnoses, markers of inflammation, weight, age, bacterial load, diversity, and relative abundance of common taxa using the Morisita-Horn beta diversity measure. (TIF) [file pone.0257838.s007.tif]

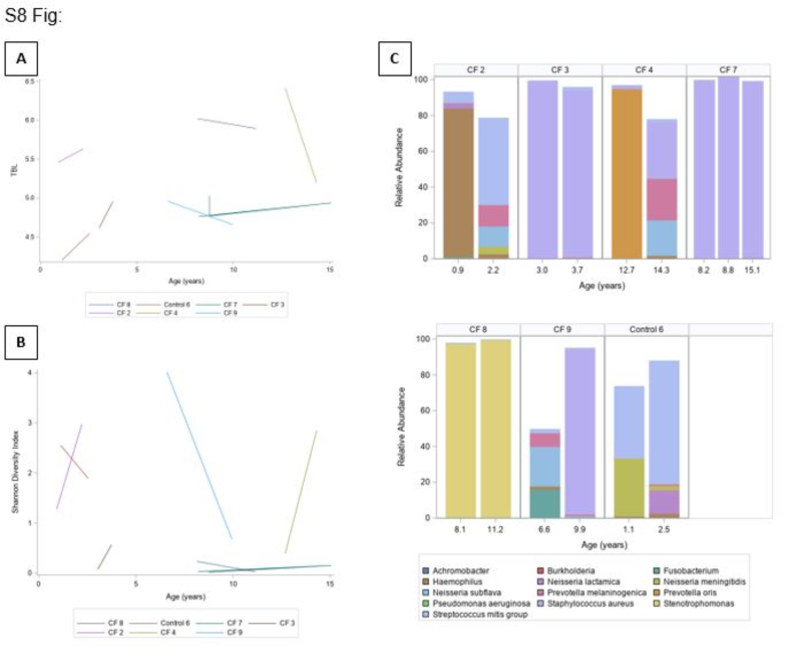

Supplement: S8 Fig — Total bacterial load (A), Shannon diversity (B) and bacterial composition (C) across the age spectrum for repeat samples from the same subjects. (TIF) [file pone.0257838.s008.tif]

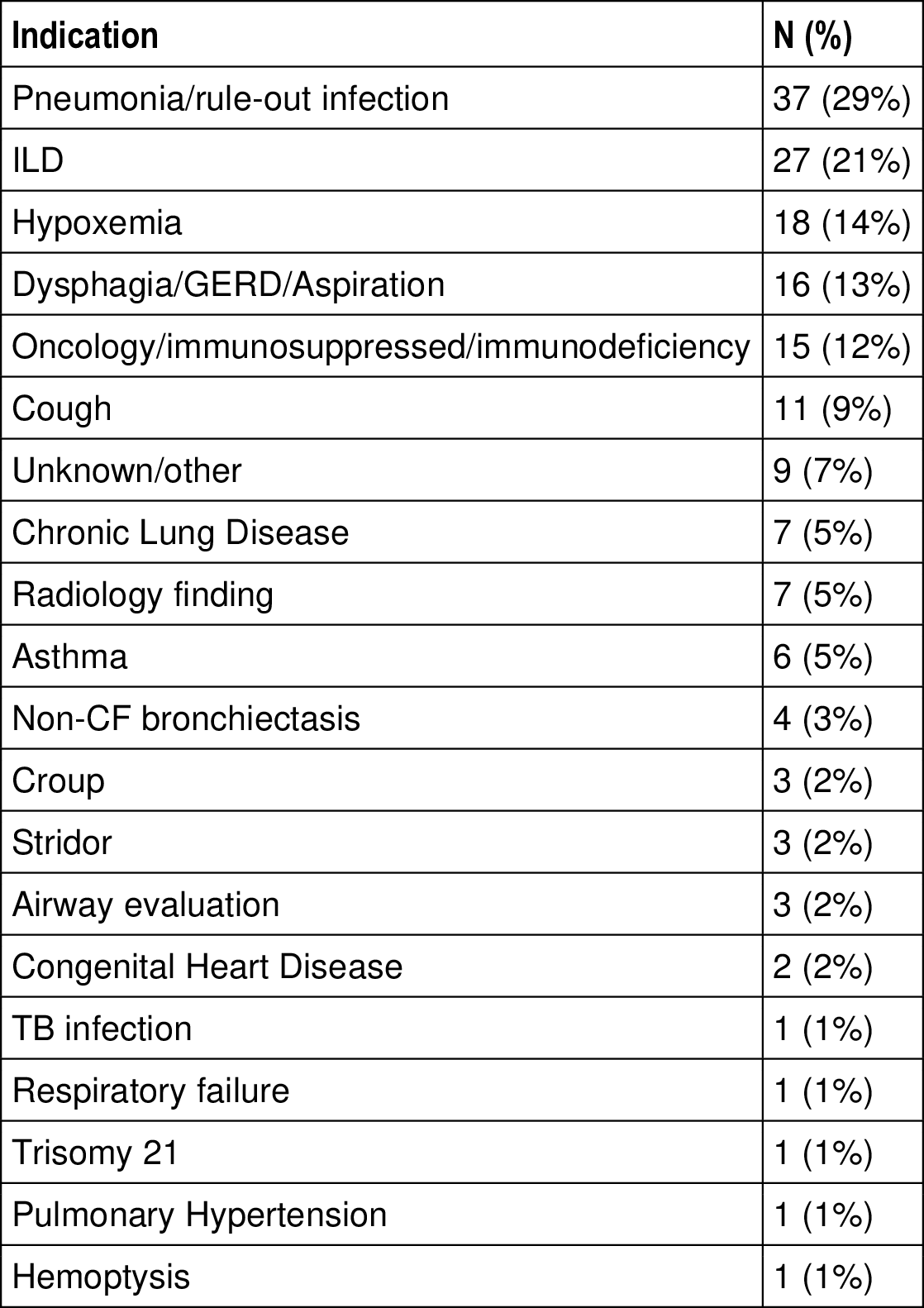

Supplement: S1 Table — Indications and Primary diagnoses of DC subjects sorted by frequency. Indication/primary diagnosis categories were not mutually exclusive with subjects falling into as many as 3 different indications. (TIF) [file pone.0257838.s009.tif]

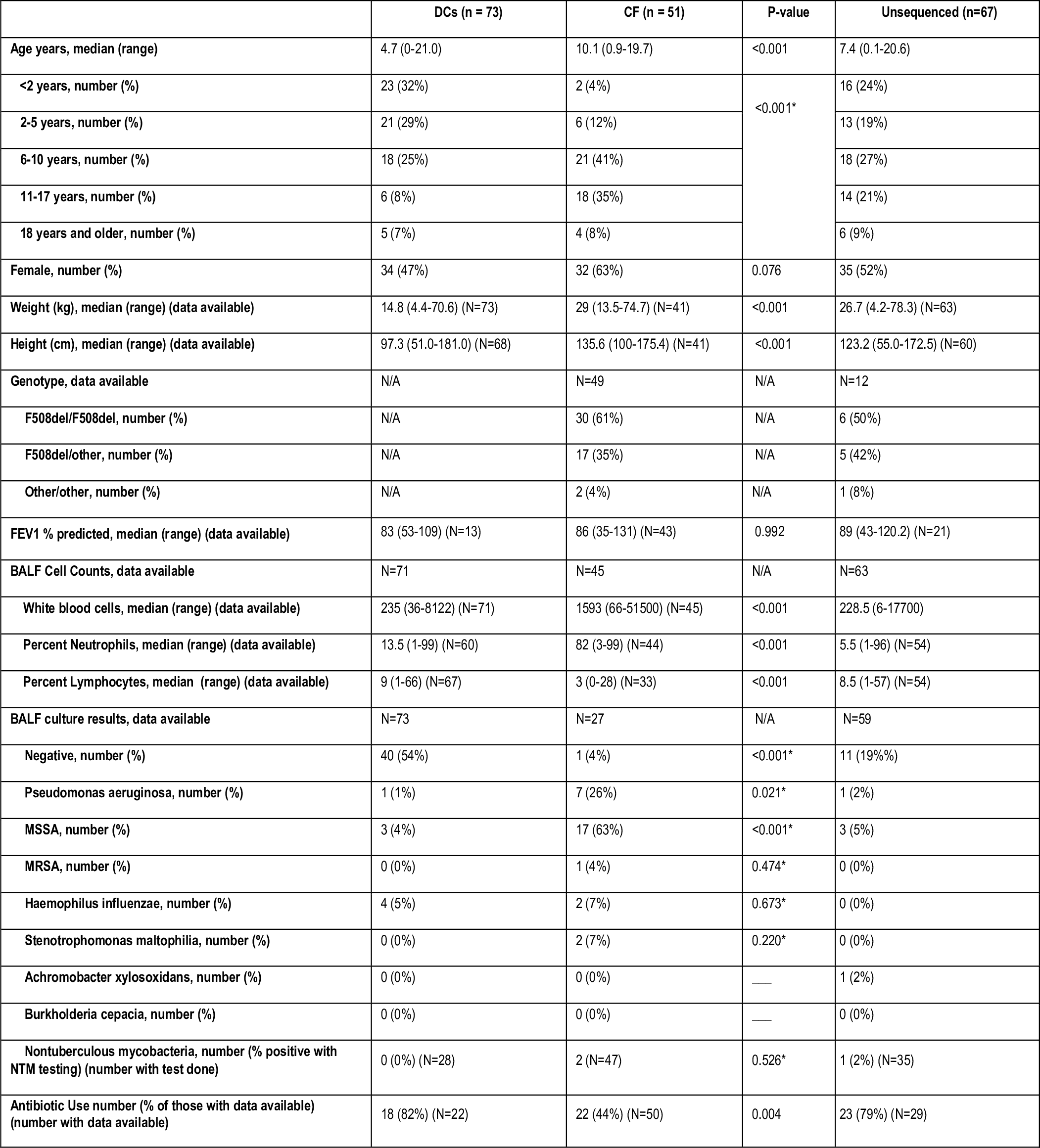

Supplement: S2 Table — Data are presented as n, median (range) or n (%), unless otherwise states.CF: Cystic fibrosis; FEV1: Forced expiratory volume in 1 s; BALF: Bronchoalveolar lavage fluid; MSSA: Methicillin-susceptible Staphylococcus aureus; MRSA. (TIF) [file pone.0257838.s010.tif]

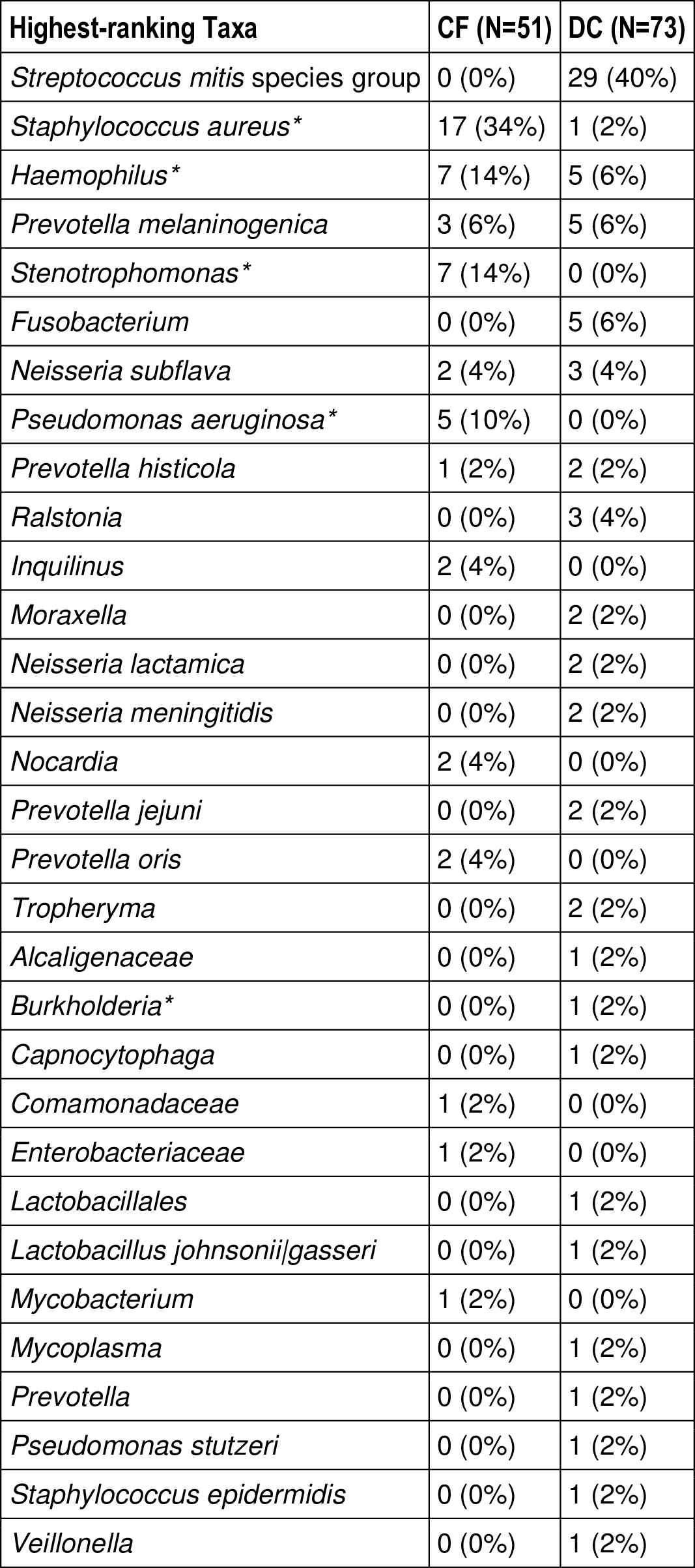

Supplement: S3 Table — Dominant taxa identified by sequencing in bronchoalveolar lavage fluid (BALF) samples from DC and cystic fibrosis (CF) subjects *Taxa associated as typical CF pathogens. Traditionally CF pathogens appear to dominate CF samples more often than DCs. (TIF) [file pone.0257838.s011.tif]

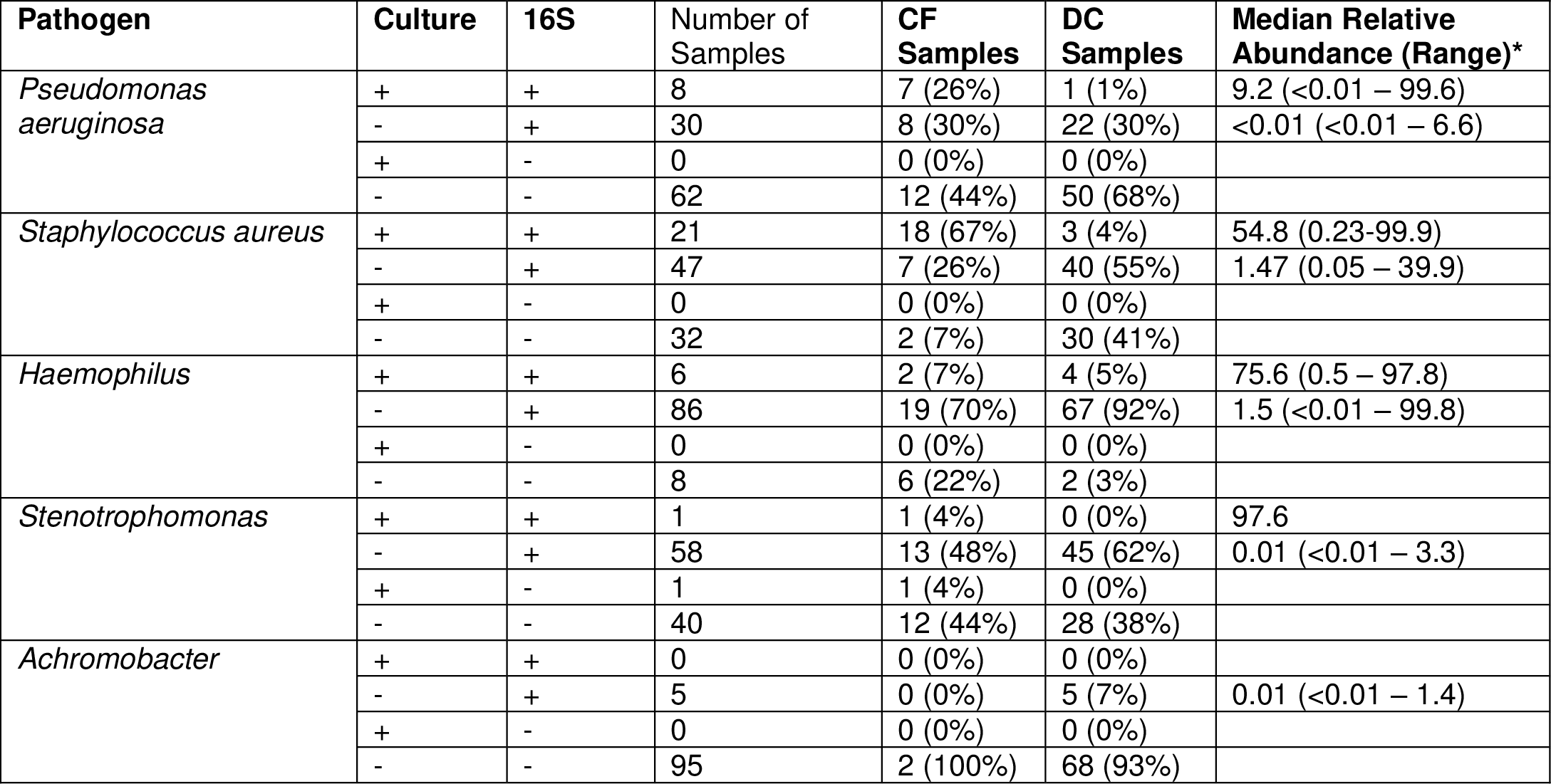

Supplement: S4 Table — Comparison of BALF culture and BALF sequencing for the identification of five common CF bacterial pathogens. + = bacteria identified either by culture or 16S, - = Not identified, *Some relative abundances are not provided as some taxa were not identified by 16S and therefore relative abundances could not be calculated. (TIF) [file pone.0257838.s012.tif]
