# Supplementary material for: Divergence of bacterial communities in the lower airways of CF patients in early childhood
Source: PLoS One. 2021 Oct 6;16(10):e0257838. doi: 10.1371/journal.pone.0257838 (PMC8494354; doi:10.1371/journal.pone.0257838)
Supplement: S1 File — (DOCX) [file pone.0257838.s013.docx]

# **Supporting Information:**

## Methods:

*DNA Extraction and Quantitative PCR*

DNA was extracted from BALF samples using the Qiagen EZ1 Advanced automated extraction platform (Qiagen, Valencia, CA, USA) per manufacturer’s instructions. Total bacterial load (TBL) was assessed using the quantitative PCR assay previously described and validated in CF airway samples [1,2]. TBL was used to estimate template requirements for amplification of the 16S rRNA gene due to the predominance of human DNA in airway samples. To reduce the impact of background, BALF samples with TBL less than 1.5x the plate background were deemed quantity not sufficient (QNS), and these extractions were not used for amplicon generation. Extraction controls (PCR water and kit buffer) were performed in parallel with BALF samples.

*High-throughput Sequencing for Microbiota Analysis*

Bacterial profiles were determined by broad-range amplification and sequence analysis of 16S rRNA as previously described [3-6]. Negative control samples were included in the sequencing analysis to assess background (S1 and S2 Figs). Amplicons generated using MiSeq compatible primers targeted ~300 base pairs of the V1/V2 variable region (27F/338R) of the 16S rRNA gene. PCR products were normalized using agarose gel densitometry, pooled, gel purified and concentrated using a DNA Clean and Concentrator Kit (Zymo, Irvine, CA). The pool of amplicons was quantified using Qubit Fluorometer 2.0 (Invitrogen, Carlsbad, CA), diluted to 4nM, and denatured with 0.2 N NaOH at room temperature. The denatured DNA was diluted to 20 pM and spiked with 10% of the Illumina PhiX control DNA prior to loading the sequencer. Illumina paired-end sequencing was performed on the MiSeq platform using a 500-cycle version 2 reagent kit.

*Analysis of Illumina Paired-end Reads*

Illumina MiSeq paired-end reads were aligned to human reference genome Hg19 with bowtie2 and matching sequences discarded [7,8] . As previously described, the remaining non-human paired-end sequences were sorted by sample via barcodes in the paired reads [6]. Sorted paired-end sequence data were deposited in the NCBI Short Read Archive under accession number PRJNA638906. The sorted paired reads were assembled using phrap [9,10], and those that did not were discarded. Assembled sequence ends were trimmed over a moving window of 5 nucleotides until average quality met or exceeded 20. Trimmed sequences with more than 1 ambiguity or shorter than 200 nt were discarded. Potential chimeras identified with Uchime (usearch6.0.203_i86linux32) [11] using the Schloss [12] Silva reference sequences were removed from subsequent analyses. Assembled sequences were aligned and classified with SINA (1.3.0-r23838) [13] using the 418,497 bacterial sequences in Silva 115NR99 [14] as reference configured to yield the Silva taxonomy. Operational taxonomic units (OTUs) were produced by clustering sequences with identical taxonomic assignments.

For the following genera*, Lactobacillus, Neisseria, Prevotella, Pseudomonas, Staphylococcus, and Streptococcus*, species identification was performed as previously reported [15]. Species identification utilized pre-computed sequence positions that identify unknown sequences within SINA/Silva designated genera. The pre-computation process indicated which species could be differentiated within a genus and which could not. In some instances, the best that could be reported was that the species were consistent with a group of species within the genus. In cases where species were not distinguishable, binomial names, when available, or accession numbers were used to identify species groups (i.e. *Streptococcus* *mitis|oralis|pneumoniae* or the *Streptococcus mitis* group), and when sequences could not be speciated into one of the species-specific OTUs, they were only assigned to that genus without any species-specification (i.e. *Streptococcus)*.

This process generated 7,438,119 sequences for 133 samples (average sequence length: 312 nt; average sample size: 55,926 sequences/sample; minimum sample size: 7,451; maximum sample size: 203,263). The median Goods coverage score was ≥ 99.86% at the rarefaction point of 7,451 sequences.

*Spline Models*

To assess the association between TBL and diversity with age by disease group, age was included in a regression model as a continuous variable and modeled using cubic B-splines that were allowed to vary by group with internal knots placed at percentiles and boundary knots placed at the extremes. The number of percentiles used for the internal knots was determined using the Bayesian Information Criterion [16]. TBL and diversity were compared at different ages between CF and DCs. Multiple comparisons were adjusted using a stepdown Holm-simulated method to control the family-wise type I error rate.

## Results:

*Repeat samples:*

The initial sample set include 206 samples, which included 15 additional repeat samples from 12 of the 191 subjects (3 DCs). Due to a long combined enrollment period of approximately 12 years, these subjects underwent multiple clinically indicated bronchoscopies over the course of the study, and multiple samples were collected. Of the 12 subjects with repeat samples, 7 had multiple samples that were successfully sequenced (S8 Fig). If the subject had successfully sequenced samples, the first sequenced sample was included in our final analyses. If no samples were sequenced, the first collected sample was included.

*Prevalence*

Taxa identified most frequently across the 124 sequenced samples, included *Veillonella* (69/124, 56%), the *Streptococcus mitis* group (65/124, 52%), *Streptococcus* (60/124, 48%), *Porphyromonas* (58/124, 47%), *Haemophilus* (58/124, 47%) and *Prevotella* (57/124, 46%). In the sequenced CF samples, the most prevalent taxon was *Staphylococcus aureus* (12/51, 24%), and, for the sequenced DC samples, the most prevalent taxa were the *Streptococcus mitis* group (58/73, 79%) and *Veillonella* (58/73, 79%).

*Highest-Ranking Taxa*

A total of 31 highest-ranking taxa were identified, 13 of which were the highest-ranking in the CF cohort and 23 of which were highest-ranking in the DCs (S3 Table). The *Streptococcus mitis* group was the most common highest-ranking taxon within the DC samples (29/73, 40%), while *Staphylococcus aureus* was the most common highest-ranking taxon in the CF samples (17/51, 34%). Taxa associated with typical CF pathogens, which included *Stenotrophomonas*, *Haemophilus*, *Pseudomonas aeruginosa*, *Staphylococcus aureus*, and *Burkholderia*, were the highest-ranking in the majority of CF samples (36/51, 71%), and the highest-ranking in only a small number of the DC samples (7/73, 10%) (p<0.001, Chi-squared test). Other notable differences between CF and DC subjects included the *Streptococcus mitis* group, which was more frequently the highest-ranking in DCs (p<0.001, Chi-squared test), and *Stenotrophomonas* (p=0.001, Chi-squared test), *Staphylococcus aureus* (p<0.001, Chi-squared test), and *Pseudomonas aeruginosa* (p=0.006, Chi-squared test)*,* which were more frequently the highest-ranking in CF samples.

*CF and DC Species Data*

Differences in the prevalent species-level taxa detected in CF and DC groups were identified. Most notably, the *Streptococcus mitis* group had increased prevalence in DC samples (p<0.001, Chi-squared test) and *Staphylococcus aureus* had increased prevalence in CF subjects (p<0.001, Chi-squared test). Differences in the RA of these two taxa across the entire age spectrum are displayed in S5 Fig. As previously described, *Staphylococcus aureus* is seen in higher abundance across the age spectrum in CF subjects when compared to DCs. The abundance of the *Streptococcus mitis* group taxon was found to be generally higher in younger subjects regardless of CF status. In CF subjects, however, there was an earlier drop in median RA observed after the age of 2.

*Clustering Analysis*

Samples were clustered into groups using the Morisita-Horn beta diversity measure (S7 Fig). This unsupervised approach results in two main clusters, a group mostly containing samples from DC subjects and one of CF samples. The DC cluster consists of two smaller clusters, one dominated by high abundant of *Streptococcus mitis* group, and one with more variability in the microbial composition. Information for other diagnoses (ILD, pneumonia, hypoxemia), inflammation, load, and diversity are displayed for samples as well. Indications for the BAL were evenly dispersed across the two smaller DC clusters and does not appear to associate with alpha diversity. The CF samples that clustered with the DCs tended to have lower inflammation, higher alpha diversity and were not dominated by traditional CF pathogens.

## References:

1. Nadkarni MA, Martin FE, Jacques NA, Hunter N. Determination of bacterial load by real-time PCR using a broad-range (universal) probe and primers set. Microbiology. 2002 Jan;148(Pt 1):257–66.
2. Zemanick ET, Wagner BD, Sagel SD, Stevens MJ, Accurso FJ, Harris JK. Reliability of quantitative real-time PCR for bacterial detection in cystic fibrosis airway specimens. PLoS One. 2010 Nov 30;5(11):e15101.
3. Laguna TA, Wagner BD, Williams CB, Stevens MJ, Robertson CE, Welchlin CW, et al. Airway Microbiota in Bronchoalveolar Lavage Fluid from Clinically Well Infants with Cystic Fibrosis [Internet]. Vol. 11, PLOS ONE. 2016. p. e0167649. Available from: <http://dx.doi.org/10.1371/journal.pone.0167649>
4. Zemanick ET, Wagner BD, Robertson CE, Ahrens RC, Chmiel JF, Clancy JP, et al. Airway microbiota across age and disease spectrum in cystic fibrosis. Eur Respir J [Internet]. 2017 Nov;50(5). Available from: <http://dx.doi.org/10.1183/13993003.00832-2017>
5. Hara N, Alkanani AK, Ir D, Robertson CE, Wagner BD, Frank DN, et al. Prevention of virus-induced type 1 diabetes with antibiotic therapy. J Immunol. 2012 Oct 15;189(8):3805–14.
6. Markle JGM, Frank DN, Mortin-Toth S, Robertson CE, Feazel LM, Rolle-Kampczyk U, et al. Sex differences in the gut microbiome drive hormone-dependent regulation of autoimmunity. Science. 2013 Mar 1;339(6123):1084–8.
7. iGenomes [Internet]. [cited 2021 Jul 9]. Available from: <https://support.illumina.com/sequencing/sequencing_software/igenome.html>
8. Langmead B, Salzberg SL. Fast gapped-read alignment with Bowtie 2. Nat Methods. 2012 Mar 4;9(4):357–9.
9. Ewing B, Green P. Base-calling of automated sequencer traces using phred. II. Error probabilities. Genome Res. 1998 Mar;8(3):186–94.
10. Ewing B, Hillier L, Wendl MC, Green P. Base-calling of automated sequencer traces using phred. I. Accuracy assessment. Genome Res. 1998 Mar;8(3):175–85.
11. Edgar RC, Haas BJ, Clemente JC, Quince C, Knight R. UCHIME improves sensitivity and speed of chimera detection. Bioinformatics. 2011 Aug 15;27(16):2194–200.\
12. Schloss PD, Westcott SL. Assessing and improving methods used in operational taxonomic unit-based approaches for 16S rRNA gene sequence analysis. Appl Environ Microbiol. 2011 May;77(10):3219–26.
13. Pruesse E, Peplies J, Glöckner FO. SINA: accurate high-throughput multiple sequence alignment of ribosomal RNA genes. Bioinformatics. 2012 Jul 15;28(14):1823–9.
14. Quast C, Pruesse E, Yilmaz P, Gerken J, Schweer T, Yarza P, et al. The SILVA ribosomal RNA gene database project: improved data processing and web-based tools. Nucleic Acids Res. 2013 Jan;41(Database issue):D590–6.
15. Mourani PM, Sontag MK, Williamson KM, Harris JK, Reeder R, Locandro C, et al. Temporal airway microbiome changes related to ventilator-associated pneumonia in children. Eur Respir J [Internet]. 2021 Mar;57(3). Available from: <http://dx.doi.org/10.1183/13993003.01829-2020>
16. Harrell FE, Harrel jrl FE. Regression Modeling Strategies: With Applications to Linear Models, Logistic Regression, and Survival Analysis. Springer Science & Business Media; 2001. 568 p.
